# Deficiency of Notch signaling in pericytes results in arteriovenous malformations

**DOI:** 10.1172/jci.insight.125940

**Published:** 2020-11-05

**Authors:** Taliha Nadeem, Wil Bogue, Bianca Bigit, Henar Cuervo

**Affiliations:** Department of Physiology and Biophysics, College of Medicine, University of Illinois at Chicago, Chicago, Illinois, USA.

**Keywords:** Angiogenesis, Vascular Biology, Cardiovascular disease, Pericytes, endothelial cells

## Abstract

Arteriovenous malformations (AVMs) are high-flow lesions directly connecting arteries and veins. In the brain, AVM rupture can cause seizures, stroke, and death. Patients with AVMs exhibit reduced coverage of the vessels by pericytes, the mural cells of microvascular capillaries; however, the mechanism underlying this pericyte reduction and its association with AVM pathogenesis remains unknown. Notch signaling has been proposed to regulate critical pericyte functions. We hypothesized that Notch signaling in pericytes is crucial to maintain pericyte homeostasis and prevent AVM formation. We inhibited Notch signaling specifically in perivascular cells and analyzed the vasculature of these mice. The retinal vessels of mice with deficient perivascular Notch signaling developed severe AVMs, together with a significant reduction in pericytes and vascular smooth muscle cells (vSMC) in the arteries, while vSMCs were increased in the veins. Vascular malformations and pericyte loss were also observed in the forebrain of embryonic mice deficient for perivascular Notch signaling. Moreover, the loss of Notch signaling in pericytes downregulated *Pdgfrb* levels and increased pericyte apoptosis, pointing to a critical role for Notch in pericyte survival. Overall, our findings reveal a mechanism of AVM formation and highlight the Notch signaling pathway as an essential mediator in this process.

## Abstract 2

Notch signaling promotes pericyte survival, and conditional loss of Notch in murine perivascular cells leads to arteriovenous malformations.

## Introduction

Arteriovenous malformations (AVMs) are focal vascular lesions where arteries shunt directly to veins without an intervening capillary bed. These lesions can occur in several organs but are particularly harmful in the brain, where they can result in seizures, stroke, and death (1). Despite their devastating consequences, the cellular and molecular basis of AVM formation is poorly understood. The sparse knowledge reported to date has addressed the contribution of the endothelium to AVM pathogenesis, while the contribution of other vascular cell types, such as vascular mural cells, remains largely unexplored.

Vascular mural cells are support cells in vessel function. There are 2 types of mural cells: pericytes and vascular smooth muscle cells (vSMCs). Pericytes and vSMCs can be distinguished by their distinct anatomical distribution, cellular morphology, and expression of certain molecular markers (2). vSMCs are present along the arteries, where they form dense layers of concentric rings. It is widely accepted that vSMCs exhibit contractile functions, mediated by proteins such as α-smooth muscle actin (α-SMA). Pericytes are commonly found on capillaries; they have rounded cell bodies with thin or helical cytoplasmic processes that extend along the abluminal surface of the vascular tube (3). Pericytes are recruited to the immature vasculature during angiogenesis to promote endothelial quiescence, vessel stability, and homeostasis (2, 4). Failure to recruit pericytes results in endothelial cell (EC) hyperplasia, blood vessel dilation, microaneurysm development, and edema (5).

In addition to abnormal vascular morphogenesis, pericyte drop out from the blood vessels is linked to human pathologies. Pericyte dysfunction or loss has been associated with diabetic retinopathy and neurodegenerative diseases (6, 7). Postmortem human brain tissues have shown that the breakdown of the blood-brain barrier (BBB) is associated with Alzheimer’s neuropathy (8). Recently, it has been reported that pericyte coverage is reduced in human brain AVMs, and pericyte reduction is most pronounced in AVMs with clinical rupture (9). Whether reduced pericyte coverage is a consequence or a cause of these vascular lesions, and which molecular pathways are involved in regulating pericyte association to the endothelium, remains unknown.

A key signaling mechanism that plays critical roles in blood vessel formation and maintenance is the highly conserved Notch pathway. The canonical Notch signaling pathway functions in various developmental processes, particularly by promoting cell differentiation (10). Mammals express 4 transmembrane Notch receptors (1–4) and 5 membrane-bound ligands (Jagged [Jag]1, Jag2, D-like ligand 1 [Dll1], Dll3, and Dll4). Signaling begins when a Notch ligand interacts with the extracellular domain of a Notch receptor of a juxtaposed cell. Ligand-receptor interaction is followed by proteolytic cleavage events to release the intracellular domain (ICD) of the receptor. Notch ICD is then trafficked to the nucleus, where it binds to the transcription factor recombination signal sequence-binding protein-JK (RBPJ) and stimulates the recruitment of Mastermind-like (MAML) and other cofactors that compose the transcriptional activation complex mediating the transcription of Notch target genes (11).

Notch signaling has been involved in mural cell differentiation and function. vSMCs express Notch2 and Notch3, and may also express Notch1 (12, 13). Pericytes have been described to express Notch1 and Notch3, as well as some levels of Notch2 (13–15). In vSMCs, Notch signaling has been widely studied and has been found to modulate vSMC differentiation, survival, and distribution on the arterial vasculature (16–18). The role of Notch in pericytes has not been clearly established. In vitro studies demonstrate that Notch activity is important for pericyte survival, for adhesion to the endothelium, and in limiting migratory and invasive behavior (19, 20). Gain-of-function and loss-of-function of Notch3 in zebrafish demonstrate that Notch3 signaling promotes pericyte proliferation and limits vascular permeability (21). However, loss of function of Notch3 in mice reports no differences on pericytes and ascribes increased vascular permeability to loss of vSMCs (17). These in vivo studies evaluate the role of Notch3 but do not consider the possible compensation of additional Notch receptors such as Notch1, which is expressed to even higher levels than Notch3 in some pericyte populations (13). Combined loss of Notch3 and haploinsufficiency of Notch1 revealed a possible role for both receptors in pericyte migration and association with the endothelium (14). However, the use of global deletion in this model makes it challenging to establish the function of Notch1 in pericytes, as opposed to other cell types (i.e., ECs) (13). In summary, there is a relative paucity of knowledge of how Notch signaling regulates pericyte function and stability in vivo.

To further understand the role of Notch signaling in pericyte function, we suppressed Notch signaling in pericytes by deleting the key transcription factor *Rbpj*, an essential component of canonical Notch signaling, using the pericyte-specific *PDGFRβ-P2A-CreER^T2^* mice (22). Here, we demonstrate that deficiency of canonical Notch signaling in pericytes results in reduced pericyte coverage and in the development of severe AVMs. Our results identify Notch signaling as a critical pathway involved in pericyte homeostasis and establish that deficiency of Notch in pericytes is sufficient to cause AVM development.

## Results

### Deficiency of Notch signaling in perivascular cells results in AVMs.

To investigate the function of Notch signaling in pericytes, we generated perivascular-specific *Rbpj* conditional KO mice by crossing *PDGFRβ-P2A-CreER^T2^* transgenic mice (22) with *Rbpj^tm1Hon^* (*Rbpj^flox^*) mice (23). *Rbpj^flox^* mice allow for Cre recombinase-dependent inactivation of Notch signaling through the deletion of *Rbpj*, a critical transcription factor regulating the expression of Notch target genes (11). *PDGFRβ-P2A-CreER^T2^* mice allow for tamoxifen-inducible expression of Cre-recombinase under the control of the platelet derived growth factor receptor-β (PDGFRβ) promoter, which is expressed in perivascular cells, and present a high recombination efficiency in the retina and brain (22). By breeding these mice together, we generated *PDGFRβ-P2A-CreER^T2^;Rbpi^fl/fl^* mutant mice, which we will refer to as Rbpj^iΔPC^, and *PDGFRβ-P2A-CreER^T2^;Rbpj^fl/+^*, which served as control mice. For our assessment of Notch signaling in pericytes during vascular development, we used the retina, one of the best-characterized tissues for angiogenesis research with a high pericyte to EC ratio (2). Inactivation of Notch signaling was prompted by treating the nursing moms with tamoxifen at P1, P2, and P3.

To verify the loss of *Rbpj* in perivascular cells of Rbpj^iΔPC^ mice, we further introduced a Cre-inducible Rosa-tdTomato reporter (Ai9) (24) in our breeding scheme. This mouse reporter will allow tdTomato expression in perivascular cells upon recombination through deletion of a STOP cassette. Retinas from Rbpj^iΔPC^ and control mice were enzymatically digested, and perivascular cells were isolated using FACS. Real-time PCR of isolated perivascular cells confirmed significantly reduced levels of *Rbpj* in this population in Rbpj^iΔPC^ mice (Supplemental Figure 1; supplemental material available online with this article; https://doi.org/10.1172/jci.insight.125940DS1).

**Figure 1:**
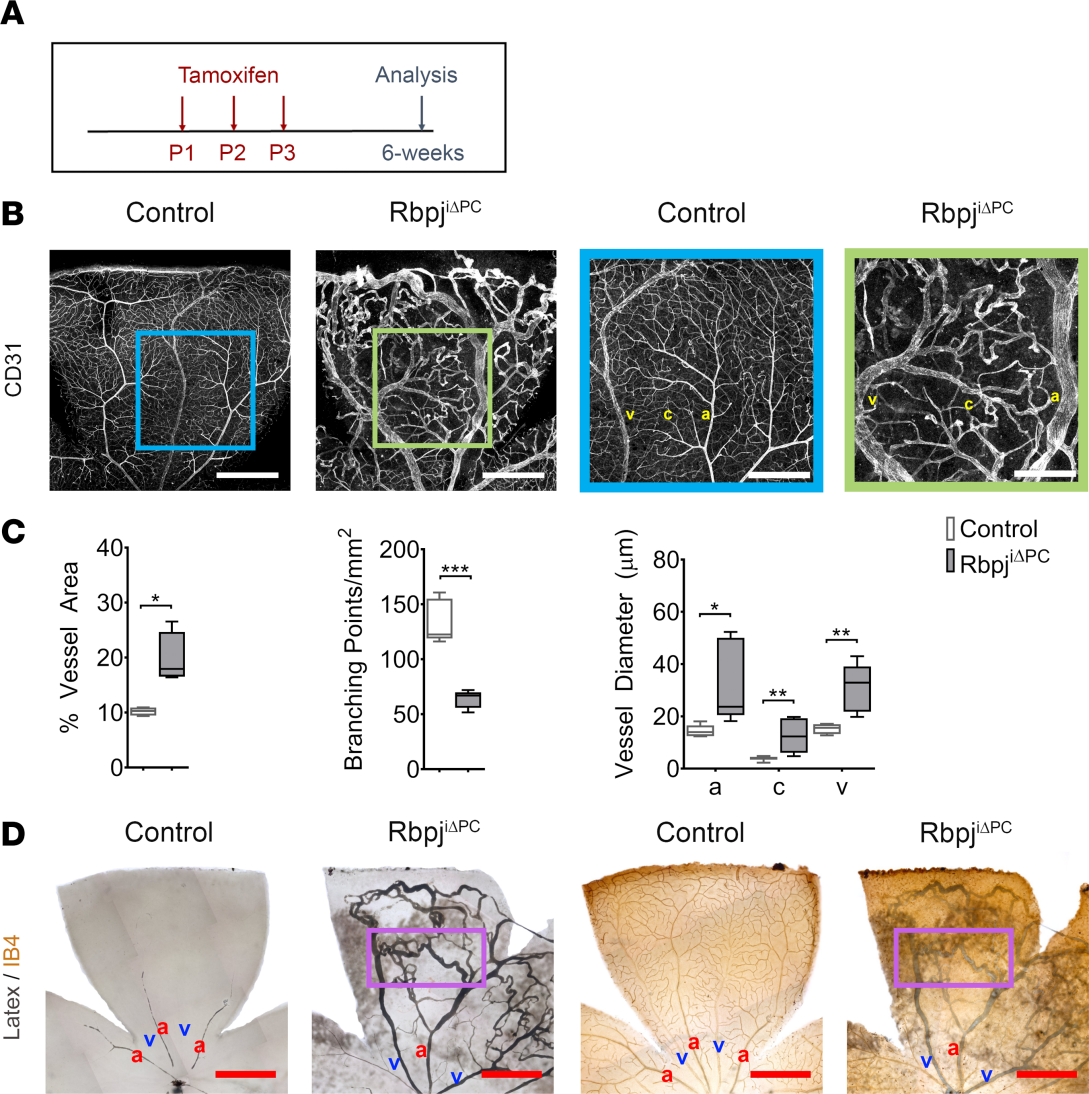
Inhibition of perivascular Notch signaling at birth results in severe AVMs. (**A**) Diagram of tamoxifen administration to postnatal control and Rbpj^iΔPC^ mice and analysis at 6 weeks. (**B**) Confocal images showing 6-week retinal vasculature stained with anti-CD31 (white). Right panels show higher magnification of boxed regions highlighting vascular malformations in the retina of Rbpj^iΔPC^ mice. Scale bars: 600 μm (left panels) and 300 μm (right panels). (**C**) Quantification of percentage of vessel area (*n* = 4), branching points/mm^2^ (*n* = 5), and vessel diameter (*n* = 6–8) in control and Rbpj^iΔPC^ mice. (**D**) Images of the vasculature perfused with blue latex compound. Perfused vessels were subsequently stained with Isolectin B4 (IB4) and detected using HRP streptavidin (*n* = 3). Boxed region highlights arteriovenous shunt. Scale bars: 600 μm. Box-and-whisker plots show median, minimum, and maximum values. Data were analyzed using unpaired 2-tailed *t* test with Welch’s correction. a, artery; c, capillary; and v, vein. *P < 0.05, **P < 0.01, ***P < 0.001.

The retinal vasculature of Rbpj^iΔPC^ was analyzed at 6 weeks of age by whole-mount immunofluorescence (Figure 1A). Isolectin B4 (IB4) or anti-CD31 were used to label the blood vessels. Severe vessel abnormalities were observed in the retinas of Rbpj^iΔPC^ compared with control mice (Figure 1B). Blood vessel area was significantly increased in Rbpj^iΔPC^ compared with control mice; however, when branching points were quantified, the number was significantly reduced (Figure 1C), indicating that an increase in the number of blood vessels was not responsible for the increased vessel area but was likely an increase in their size. Consistent with these observations, we observed that vessel diameter was significantly higher in mutant mice compared with controls (Figure 1C).

Enlarged capillary diameter highlighted wide connections between arteries and veins. Such enlarged connections are a hallmark of AVMs, where arteries shunt directly into the veins without the narrowing of an intervening capillary bed. To evaluate whether the observed connections were forming arteriovenous shunts, we perfused Rbpj^iΔPC^ mice and controls with a blue latex compound. This compound was injected through the heart in the left ventricle; in the case of healthy (control) mice, it filled the arteries and arterioles, but its density prevented it from penetrating into the small capillary vessels and the venous compartment (Figure 1D). In the case of Rbpj^iΔPC^ mice, the latex compound traveled through the enlarged connections, filling both the arterial and venous compartments of the capillary bed, demonstrating the presence of AVMs (Figure 1D). At this time point, 92% of Rbpj^iΔPC^ mice (22 of 24) exhibited retinal AVMs, while there were no AVMs present in the control mice population.

Taken together, these results demonstrate that insufficient Notch signaling in perivascular cells results in a loss of blood vessel number but in increased vascular area and diameter, resulting in the formation of AVMs.

### Deficiency of Notch signaling in perivascular cells results in reduced pericyte coverage and abnormal vSMC distribution.

**Figure 2:**
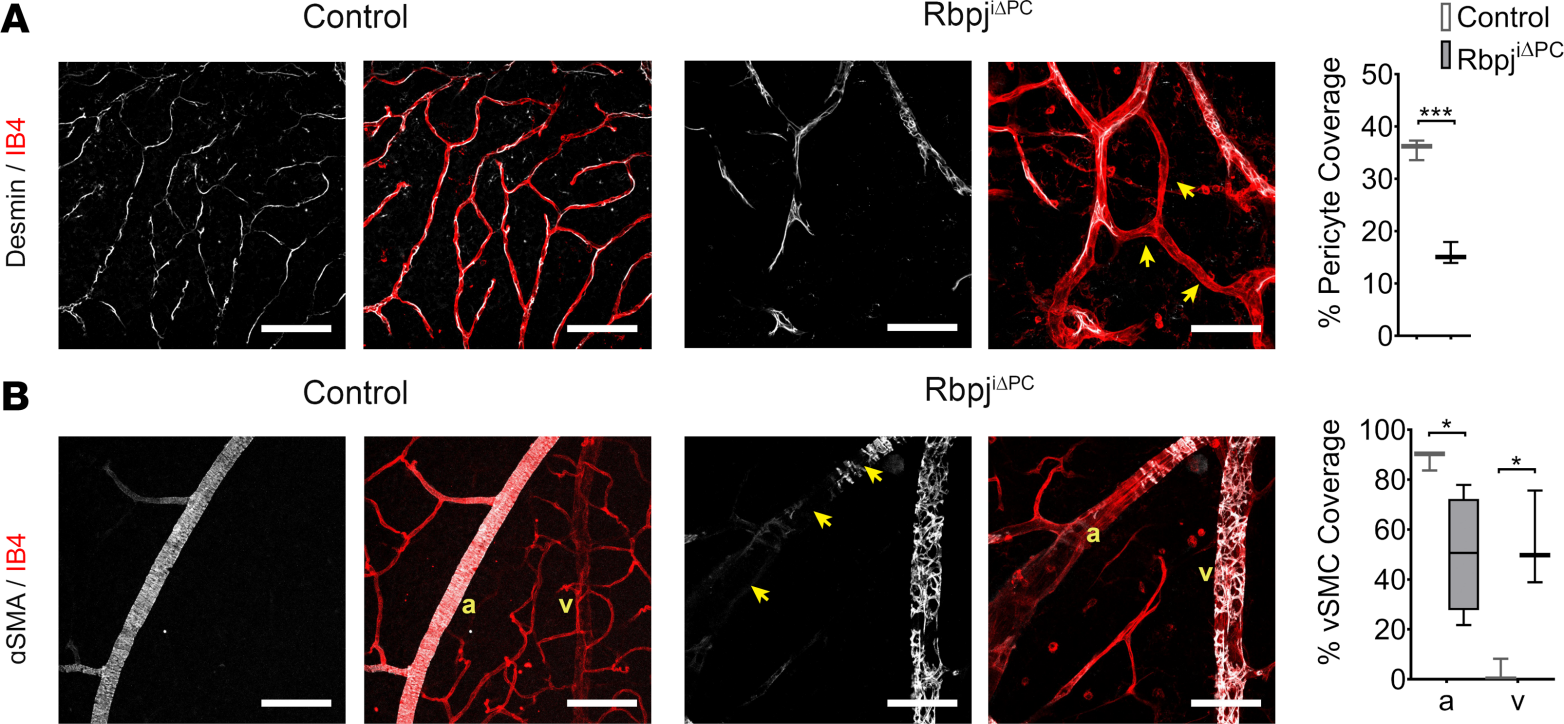
Rbpj^iΔPC^ mice present significantly reduced pericyte coverage and irregular vSMC distribution at 6 weeks. (**A**) Images and quantification of retinal vasculature of control and Rbpj^iΔPC^ mice stained with Isolectin B4 (IB4, red) and anti-Desmin (white). Note that enlarged IB4^+^ capillaries of Rbpj^iΔPC^ mice are associated with reduced Desmin^+^ pericyte coverage, indicated by the arrows. Scale bars: 100 μm. Quantification of percentage of pericyte coverage (*n* = 3) in control and Rbpj^iΔPC^ mice. (**B**) Images and quantification of vascular smooth muscle cells (vSMCs). IB4 (red) labels the endothelium and α-SMA (white) labels vascular smooth muscle cells (vSMCs). Note IB4^+^ arteries have reduced α-SMA^+^ vSMCs, as indicated by the arrows. IB4^+^ veins of Rbpj^iΔPC^ are associated with increased α-SMA^+^ perivascular cells. a, artery; v, vein. Scale bars: 100 μm. Quantification of percentage of vSMC coverage (*n* = 3–4) in control and Rbpj^iΔPC^ mice. Box-and-whisker plots show median, minimum, and maximum values. Data were analyzed using unpaired 2-tailed *t* test with Welch’s correction. *P < 0.05, ***P < 0.001.

To gain insight into whether the vascular abnormalities observed in retinas from Rbpj^iΔPC^ mice were associated with changes in perivascular cell distribution, we evaluated pericyte and vSMC coverage of the endothelium. Pericytes were identified as Desmin-expressing cells in the capillary vessels. Analysis of pericyte coverage showed a significant reduction in the percentage of vessel area covered by pericytes in Rbpj^iΔPC^ mice (Figure 2A). Analysis of vSMCs, identified by ɑ-SMA, revealed a marked reduction in vSMC coverage on arteries of Rbpj^iΔPC^ retinas compared with control, with extensive gaps between adjacent vSMCs (Figure 2B). Moreover, we also noticed a remarkable increase in ɑ-SMA expression in mural cells on the veins, which normally express low levels ɑ-SMA (Figure 2B). Together, these results show that Notch signaling is critical to maintain the correct distribution of mural cell populations through the vasculature.

### Perivascular Notch signaling in early angiogenesis.

Despite the fact that the cause of AVMs is mostly unknown, much of the current evidence points to a multifactorial etiology of genetic mutations and angiogenic stimulation (25). Hence, we hypothesized that AVMs in Rbpj^iΔPC^ mice were arising due to defects in angiogenesis and/or in the maturation of the immature vasculature into distinct arteries, capillaries, and veins.

**Figure 3:**
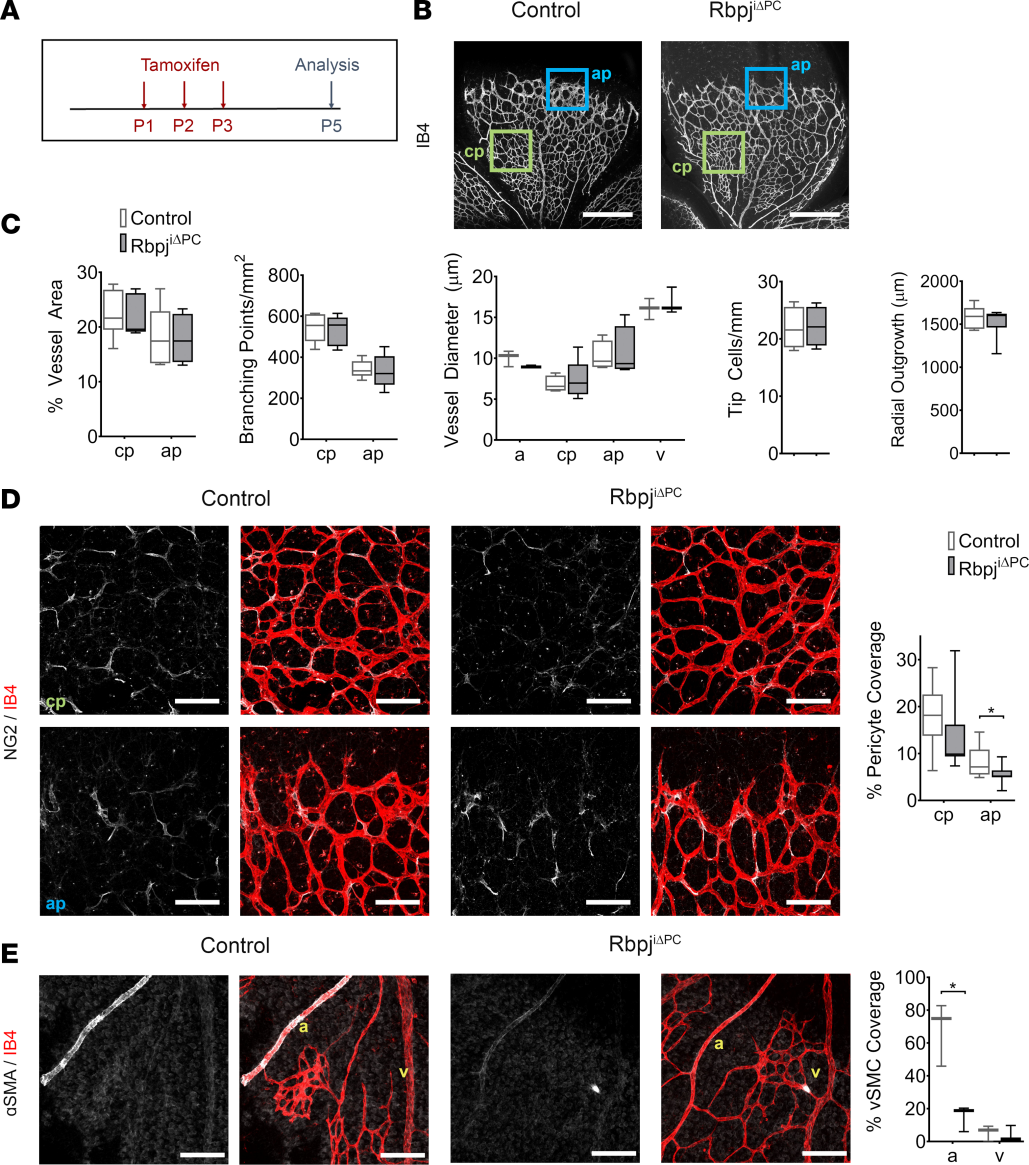
Decreased pericyte coverage in Rbpj^iΔPC^ mice at P5. (**A**) Diagram of tamoxifen administration to postnatal control and Rbpj^iΔPC^ mice and analysis at P5. (**B**) Confocal images showing P5 retinal vasculature stained with Isolectin B4 (IB4, white). Blue box represents the angiogenic plexus (ap), and green box represents the capillary plexus (cp) utilized for analysis. Scale bars: 450 μm. (**C**) Quantification of percentage of vessel area (*n* = 5–6), branching points/mm^2^ (*n* = 5), vessel diameter (*n* = 3–5), tip cells/mm^2^ (*n* = 4), and radial outgrowth (*n* = 6–8) in control and Rbpj^iΔPC^ mice. (**D**) High-magnification confocal images and quantification of NG2^+^ pericytes. NG2 (white) labels the pericytes and IB4^+^ (red) labels the vessels in the capillary and angiogenic plexus. Scale bars: 100 μm. Quantification of percentage of pericyte coverage (*n* = 9–11). (**E**) IB4 (red) staining highlights the arteries and veins and α-SMA (white) represents the vascular smooth muscle cells (vSMCs). Scale bars: 100 μm. Quantification of percentage of vSMC coverage (*n* = 3) reveals reduced arterial coverage. Box-and-whisker plots show median, minimum, and maximum values. Data were analyzed using unpaired 2-tailed *t* test with Welch’s correction. a, artery; v, vein. *P < 0.05.

To investigate whether AVMs were developing because of a defect in blood vessel formation, we analyzed the vasculature of Rbpj^iΔPC^ and control mice at P5. At this age, the retinal plexus grows rapidly through sprouting angiogenesis (26). As with previous analyses, Rbpj^iΔPC^ and control mice received tamoxifen at P1, P2, and P3 (Figure 3A). At P5, whole-mount retinas were immunostained for ECs using IB4, for pericytes using anti–Neuron-glial antigen 2 (NG2), and for vSMCs using anti–α-SMA. Analysis of the vascular plexus did not reveal any differences in the vessel area, vessel diameter, number of branching points, number of tip cells, or radial outgrowth (Figure 3, B and C), indicating that loss of Notch in pericytes did not alter angiogenesis at this early stage. Analysis of pericyte coverage showed a significant reduction of pericyte coverage in the angiogenic front of Rbpj^iΔPC^ mice compared with control, while no differences were observed in the area closer to the optic nerve, where vessels are more mature (Figure 3D). Evaluation of vSMCs showed that ɑ-SMA–expressing cells were significantly reduced in the arteries of Rbpj^iΔPC^ compared with control (Figure 3E); however, no presence of ɑ-SMA in cells surrounding the veins was detected (Figure 3E).

Previous literature suggests that pericyte depletion at the angiogenic front plexus leads to increased EC expansion (27). Although we observed no significant vascular abnormalities at P5, we investigated the endothelium further to see whether Rbpj^iΔPC^ mice presented increased EC proliferation. For this purpose, we injected mice with the thymidine analog EdU and labeled EC nuclei immunostaining for early growth response factor-1 (Erg1) (Supplemental Figure 2A). We found no significant differences in EC proliferation in retinas from Rbpj^iΔPC^ mice compared with control (Supplemental Figure 2B). Considering that the lack of pericytes also leads to increased EC size (28), we next evaluated EC morphology by immunostaining against vascular endothelial cadherin (VE-Cadherin). Similarly to EC proliferation, no changes in EC size were observed at this time (Supplemental Figure 2C).

Altogether, our data indicate that Notch signaling in pericytes does not regulate early sprouting angiogenesis but is necessary for mural cell coverage on capillaries and arteries.

### Deficiency of Notch signaling in perivascular cells impairs vascular remodeling.

Since perivascular Notch did not seem to regulate the early angiogenic response, we postulated that perhaps it had a function in regulating vessel remodeling/maturation. For this purpose, we evaluated mice at P10, when the superficial vasculature of the retina is undergoing severe remodeling and the blood vessels from the immature angiogenic plexus are establishing their final caliber and specializing into arteries, mature capillaries, and veins (26). Rbpj^iΔPC^ and control mice received tamoxifen at P1–P3 (Supplemental Figure 3A). Retinas were isolated at P10 and immunostained for endothelial and mural cell markers (Supplemental Figure 3, B, D, and E). We observed a decrease, although not significant, in branching points in the peripheral capillaries of Rbpj^iΔPC^ retinas when compared with control (Supplemental Figure 3C). This was accompanied by a significant increase in capillary and arterial diameter in retinas from Rbpj^iΔPC^ mice compared with control mice (Supplemental Figure 3C). Of note, we did not detect significant changes in vein diameter (Supplemental Figure 3C).

When we assessed pericytes using anti-Desmin, we observed a significant decrease in pericyte coverage (Supplemental Figure 3D) in retinas from Rbpj^iΔPC^ mice compared with control mice. Analysis of vSMC revealed apparent gaps in coverage of ɑ-SMA–expressing vSMC on arteries (Supplemental Figure 3E). Additionally, we observed a significant increase in the coverage of ɑ-SMA–expressing mural cells on the veins (Supplemental Figure 3E).

At P10, the dense and immature primitive vasculature undergoes an extensive remodeling process marked by a decline in vessel diameter and vascular area. However, in the absence of pericytes in the retinas from Rbpj^iΔPC^, the vessels appeared severely enlarged compared with control mice. To determine the cause of vessel dilation in retinas from Rbpj^iΔPC^ mice, we assessed EC proliferation and size. We did not observe significant changes in EC proliferation in the capillaries of Rbpj^iΔPC^ mice compared with control (Supplemental Figure 4, A and B). However, there was a significant increase in EC size in both the middle and distal capillaries of mutant mice compared with controls (Supplemental Figure 4C). These data suggest that the observed vessel enlargement in Rbpj^iΔPC^ retinas is a consequence of increased EC size.

**Figure 4:**
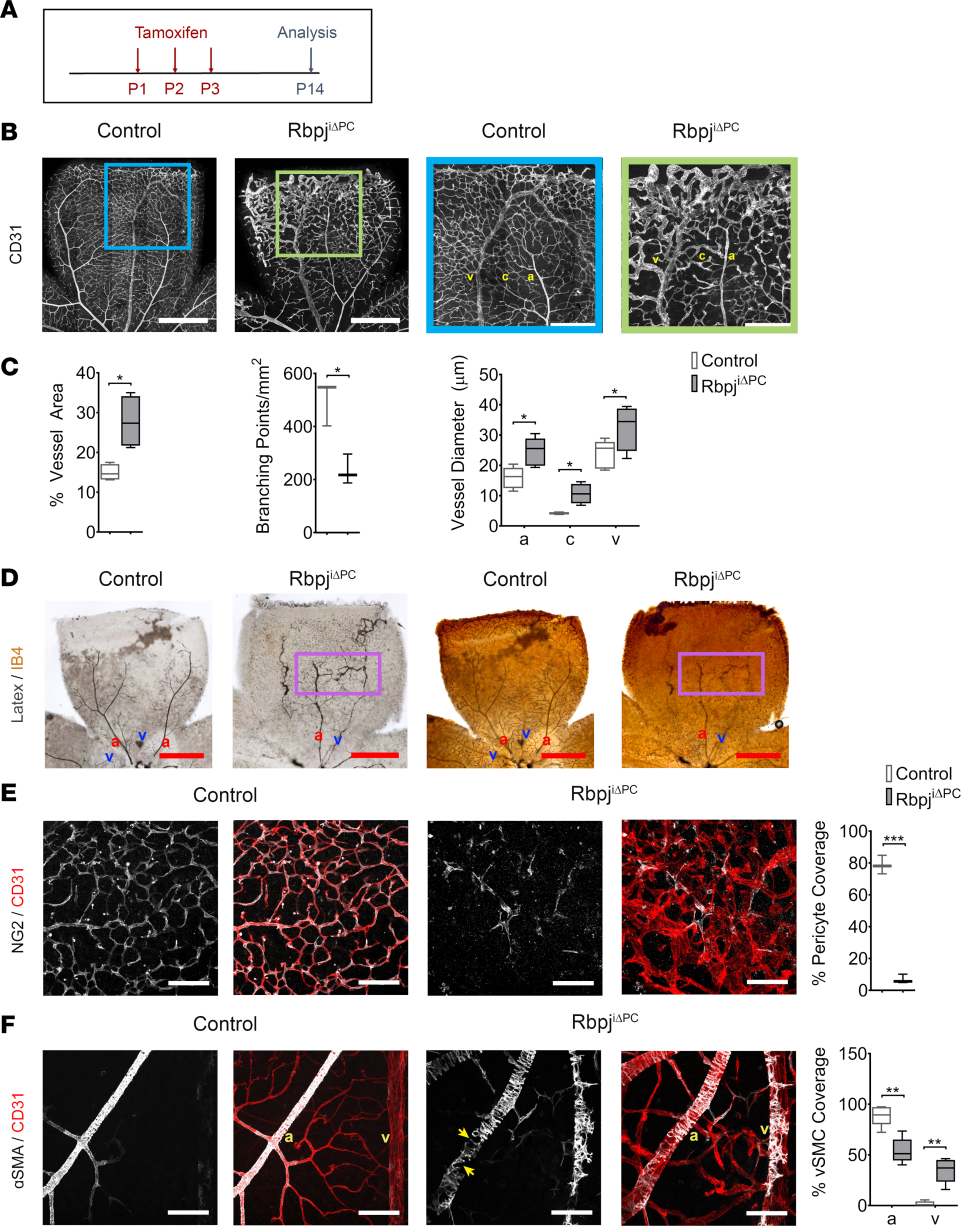
AVMs following inhibition of Notch signaling in pericytes at P14. (**A**) Diagram of tamoxifen administration to postnatal control and Rbpj^iΔPC^ mice and analysis at P14. (**B**) Confocal images showing P14 retinal vasculature stained with anti-CD31 (white). Right panels show higher magnification of boxed regions, demonstrating vascular abnormalities in the retina of Rbpj^iΔPC^ mice. Scale bars: 600 μm (left panels) and 300 μm (right panels). (**C**) Quantification of percentage of vessel area (*n* = 4), branching points/mm^2^ (*n* = 3), and vessel diameter (*n* = 4–6) in control and Rbpj^iΔPC^ mice. (**D**) Images of vasculature perfused with blue latex compound. Perfused vessels were subsequently stained with IB4 and detected using HRP streptavidin. Boxed region highlights AV shunt. Scale bars: 200 μm. (**E**) High-magnification confocal images of anti-NG2 (white) and anti-CD31 (red) staining of pericytes and the capillaries, respectively. Scale bars: 100 μm. Quantification of percentage of pericyte coverage (*n* = 3) in control and Rbpj^iΔPC^ mice. (**F**) CD31 (red) highlights the arteries and veins, and α-SMA (white) represents vascular smooth muscle cells (vSMCs). Note CD31^+^ arteries have reduced α-SMA^+^ vSMCs, as indicated by arrows. Scale bars: 100 μm. Quantification of percentage of vSMC coverage (*n* = 5) in control and Rbpj^iΔPC^ mice. Box-and-whisker plots show median, minimum, and maximum values. Data analyzed using unpaired 2-tailed *t* test with Welch’s correction. a, artery; c, capillary; and v, vein. *P < 0.05, **P < 0.01, ***P < 0.01.

By P14, a later stage of vascular maturation in the retina, we observed a pronounced increase in vessel area accompanied by a significant reduction in the number of branching points (Figure 4, A–C) in mutant mice. Additionally, analysis of vessel diameter revealed significantly enlarged blood vessels, including enlargement of the veins (Figure 4, B and C). Enlargement of all blood vessels at this developmental stage prompted us to check for AVMs using blue latex compound (Figure 4D). AVMs were observed in 100% of Rbpj^iΔPC^ mice (18 of 18) but not in the control group.

Evaluation of NG2 immunostaining showed a marked reduction in pericyte coverage in retinas from Rbpj^iΔPC^ compared with control mice (Figure 4E). Furthermore, there were substantial gaps in vSMC coverage on the arteries by P14 and a robust increase in α-SMA–expressing mural cells on the veins (Figure 4F).

Taken together, our data show a severe mural cell and vascular dysfunction by P10 and P14. Our results point to a critical role of Notch signaling in pericytes during the remodeling of the immature vasculature into the mature, fine caliber vessels through regulation of EC cell size.

### Perivascular Notch deficiency results in impaired endothelial barrier and hemorrhage.

Pericyte coverage is critical for endothelial barrier maturation and function (2), and models of pericyte loss present compromised endothelial cell–cell junctions (28). To determine if the loss of perivascular Notch signaling affected endothelial cell–cell junctions, we labeled EC junctions with VE-Cadherin. In the retinas of Rbpj^iΔPC^ mice, VE-Cadherin staining showed discontinuous adherens junctions, suggesting compromised barrier function at P10 (Supplemental Figure 4D). Consistent with this morphological defect, we detected retinal hemorrhages in 20% of Rbpj^iΔPC^ mice (2 of 10) at P10, and 40% of Rbpj^iΔPC^ mice (8 of 20) at P14 (Supplemental Figure 4E). These findings indicate that perivascular Notch signaling is crucial for vessel barrier stability.

### Deficiency of Notch signaling in vSMCs does not result in AVM formation.

PDGFRβ is expressed in both pericytes and SMCs, and while the mouse line we are using to impair Notch signaling preferentially targets pericytes (29), it might also be affecting Notch signaling in vSMCs (30). To determine whether the observed outcomes after impairing Notch signaling were due to the effect on pericytes or on vSMCs, we used a mouse line targeting vSMCs and not pericytes: *Acta2-CreER^T2^* (31). We first analyzed the efficiency of the Cre-recombinase activity of *Acta2-CreER^T2^* mice by crossing it with Rosa-tdTomato reporter mice. Tamoxifen was administered to the nursing mom at P1–P3 (Supplemental Figure 5A), and at P5, we evaluated tdTomato protein expression together with anti–ɑ-SMA to label vSMCs. Expression of tdTomato was observed in ɑ-SMA–expressing cells lining the arteries with a recombination efficiency of 77.09% ± 2.89%. (Supplemental Figure 5B).

We next crossed *Acta2-CreER^T2^* mice with *Rbpj^flox^* mice to generate *Acta2-CreER^T2^*;R*bpj^fl/fl^* (Rbpj^iΔSMC^) and control littermates (*Acta2-CreER^T2^*;R*bpj^fl/+^*). To verify a successful suppression of Notch signaling in vSMCs using Rbpj^iΔSMC^ mice, we further crossed them with the *CBF:H2B-Venus* mouse line (32). *CBF:H2B-Venus* mice express a nuclear-fluorescent protein under the control of RBPJ-responsive elements. We assessed Venus expression in vSMCs from Rbpj^iΔSMC^:*CBF:H2B-Venus* mice at P5 following tamoxifen treatment at P1–P3 (Supplemental Figure 5C). We found that reporter expression in arterial vSMCs was significantly reduced in Rbpj^iΔSMC^ mice when compared with control littermates (Supplemental Figure 5, D and E), validating successful recombination of *Rbpj* and reduction in Notch signaling in vSMCs of Rbpj^iΔSMC^ mice.

We then proceeded to analyze control and Rbpj^iΔSMC^ mice for vascular abnormalities. Rbpj^iΔSMC^ and control mice received tamoxifen at P1–P3, and retinas were harvested for analysis at P14 — the earliest time point we had detected AVM formation in Rbpj^iΔPC^ mutants. Retinas were immunostained with endothelial and perivascular markers. Examination of the vascular plexus revealed no differences in the vessel area, vessel diameter, or pericyte coverage (Supplemental Figure 6, A and B). These data indicate that deficiency of Notch signaling in vSMCs does not recapitulate the vascular malformations observed in Rbpj^iΔPC^, and they point to Notch signaling in pericytes, rather than vSMCs, as the key mediator in inducing pericyte dysfunction and AVM formation.

### Deficiency of Notch signaling in pericytes results in blood vessel regression.

Previous analysis of P14 and 6-week retinas revealed a significantly reduced number of branching points in Rbpj^iΔPC^ retinas compared with controls, indicating a reduced number of vessel segments in these mice. Because we did not see any defects in angiogenesis at P5, we hypothesized that the reduction in branching points may be due to regression of newly formed vessels.

**Figure 5:**
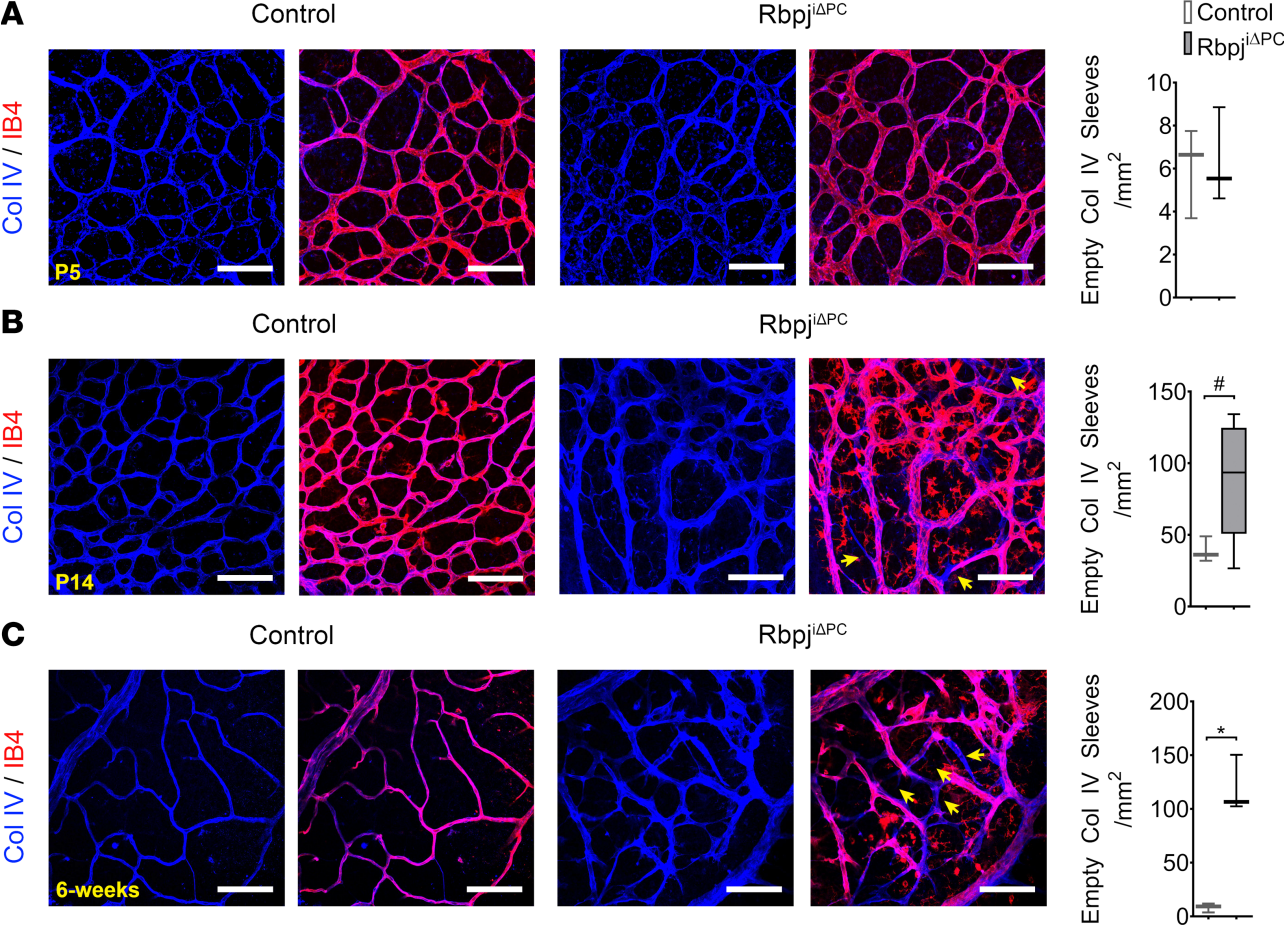
Increased vessel regression in Rbpj^iΔPC^ mice. (**A**–**C**) High-magnification confocal images of retinal vasculature stained with Isolectin B4 (IB4, red) and anti–Collagen IV (Col IV, blue) at P5, P14, and 6 weeks. Arrows highlight empty Col IV sleeves (Col IV^+^, IB4^–^). Scale bars: 100μm. Quantification of the number of empty Col IV sleeves/mm^2^ area of P5 (*n* = 3), P14 (*n* = 3-5), and 6 weeks (*n* = 3). Unpaired 2-tailed *t* test with Welch’s correction. *P < 0.05. ^#^*P* = 0.0526.

Collagen IV constitutes part of the basement membrane of blood vessels and is deposited around new vascular branches as they are formed (33, 34). When a vessel branch regresses, it leaves behind an empty basement membrane sleeve as a footprint indicative of its regression. To analyze the degree of vascular regression, we immunostained the retinas of Rbpj^iΔPC^ and control mice for ECs and Collagen IV at P5, P14, and 6 weeks. Analysis of P5 retinas showed no significant differences in the number of empty collagen sleeves between Rbpj^iΔPC^ and control mice (Figure 5A). Analysis at P14 revealed an increase in the number of empty collagen sleeves; however, this increase was nonsignificant (Figure 5B). Analysis at 6 weeks revealed a significant increase in the number of Collagen IV empty sleeves present in mutants compared with controls (Figure 5C).

To further understand whether vessel regression was due to poor vessel perfusion, we injected 6-week-old control and Rbpj^iΔPC^ mice with fluorescent Tomato Lectin in the systemic circulation before retinal dissection. We found that blood vessels of Rbpj^iΔPC^ retinas were poorly perfused compared with controls, possibly explaining the marked increase in vessel regression observed in these mice (Supplemental Figure 7).

### Loss of canonical Notch signaling and not Rbpj-mediated Notch-independent effects causes loss of pericytes and AVM formation.

We have targeted *Rbpj*, a well-established mediator of canonical Notch signaling (10), to investigate the effects of impaired Notch signaling. However, Notch-independent Rbpj-mediated effects have been reported in the literature (15, 35, 36). To verify that the phenotypes observed in Rbpj^iΔPC^ mice were a result of disrupting canonical Notch signaling and not Rbpj-mediated Notch-independent effects, we used *PDGFRβ-P2A-CreER^T2^* mice to overexpress a dominant negative form of MAML in perivascular cells (37). Expression of dominant negative MAML (dnMAML) also inhibits canonical Notch signaling by preventing the formation of the Notch transcription complex (11), but it does not affect alternative functions of Rbpj.

*PDGFRβ-P2A-CreER^T2^;dnMAML^fl/fl^* (dnMAML^iOEPC^) and littermate controls (*PDGFRβ-P2A-CreER^T2^;dnMAML^+/+^*) were treated with tamoxifen at P1, P2, and P3 (Supplemental Figure 8A). At 6 weeks, retinas were harvested and immunostained for endothelial and perivascular markers. Evaluation of the vasculature showed enlarged tortuous blood vessels forming connections between arteries and veins in dnMAML^iOEPC^ mice compared with control mice and a reduction in pericyte and vSMC coverage, recapitulating the phenotype observed in Rbpj^iΔPC^ mice at this time point (Supplemental Figure 8B). We found that 80% of dnMAML^iOEPC^ mice (4 of 5) exhibited retinal AVMs, while there were no AVMs present in the control mice.

These findings suggest that pericyte dysfunction and the subsequent vascular abnormalities and AVMs observed in Rbpj^iΔPC^ mice are due to impaired canonical Notch signaling and not Rbpj-mediated Notch-independent signaling mechanisms.

Previous literature documents Notch1 and Notch3 as the main Notch receptors expressed in brain pericytes (13, 14). To further establish a function for canonical Notch signaling in pericytes, we crossed *PDGFRβ-P2A-CreER^T2^* mice with the previously published *Notch1^flox^* mice (38) and the newly generated *Notch3^flox^* mice. To verify efficient loss of *Notch3* using the newly generated *Notch3^flox^* line, we crossed it with the *ROSA26-CreERT^2^* (39) mouse line to generate *ROSA26-CreER^T2^;Notch3^fl/fl^* mice and evaluated the levels of Notch3 in the retinas of P14 mice treated with tamoxifen at P1–P3. Notch3 was detected in C57BL/6J mice but not in tamoxifen-treated *ROSA26-CreER^T2^;Notch3^fl/fl^* or *Notch3*-null mice (40) (Supplemental Figure 9).

From crosses using both *Notch1^flox^* and *Notch3^flox^* mice, we obtained genotypes with varying levels of Notch1 and Notch3 deficiencies. Mutant mice include *PDGFRβ-P2A-CreER^T2^;Notch1^fl/fl^*;*Notch3^fl/+^* (Notch1^iΔPC^;Notch3^iΔPC/+^ mice), *PDGFRβ-P2A-CreER^T2^;Notch1^+/+^*;*Notch3^fl/fl^* (Notch1^+/+^;Notch3^iΔPC^ mice), *PDGFRβ-P2A-CreER^T2^;Notch1^fl/+^*;*Notch3^fl/fl^* (Notch1^iΔPC/+^;Notch3^iΔPC^ mice), and double-homozygous mutant mice *PDGFRβ-P2A-CreER^T2^;Notch1^fl/fl^*;*Notch3^fl/fl^* (Notch1^iΔPC^;Notch3^iΔPC^). For control mice, we used *PDGFRβ-P2A-CreER^T2^;Notch1^fl/+^*;*Notch3^fl/+^*, which are heterozygous for Notch1 and Notch3 and annotated as Notch1^iΔPC/+^;Notch3^iΔPC/+^.

We induced loss of both *Notch1* and *Notch3* in pericytes through tamoxifen delivery at P1–P3 (Supplemental Figure 10A). At P14, we observed no overt changes in mural cell coverage and vascular morphogenesis in the retinas from Notch1^iΔPC/+^;Notch3^iΔPC/+^ mice (control). Retinas from Notch1^iΔPC^;Notch3^iΔPC/+^ exhibited a phenotype similar to controls; however, Notch1^+/+^;Notch3^iΔPC^ mice and Notch1^iΔPC/+^;Notch3^iΔPC^ displayed an increase in vessel diameter and a reduction in mural cells on capillaries and arteries. The double homozygous mutant mice for Notch1 and Notch3 presented the most severe decrease in pericyte and arterial vSMCs, an increase in α-SMA expression on vein mural cells, and overall, altered retinal vascular morphogenesis resembling that observed in P14 Rbpj^iΔPC^ retinas (Supplemental Figure 10, B and C). We observed AVMs in 17% of Notch1^iΔPC^;Notch3^iΔPC/+^ mice (1 of 6), 0% of Notch1^+/+^;Notch3^iΔPC^ mice (0 of 2), 100% of Notch1^iΔPC/+^;Notch3^iΔPC^ mice (8 of 8), and 100% of Notch1^iΔPC^;Notch3^iΔPC^ mice (8 of 8). We did not observe any AVMs in control (Notch1^iΔPC/+^;Notch3^iΔPC/+^ mice) mice (0 of 7).

Together, these data establish that the loss of canonical Notch signaling through Notch1 and Notch 3 receptors mediates pericyte loss and AVM development.

### Embryonic, but not postnatal, deficiency of perivascular Notch signaling leads to vascular malformations in the brain.

To determine whether inactivation of Notch signaling results in vascular abnormalities in other organs, we examined H&E-stained sections of the brain, heart, liver, and lung of Rbpj^iΔPC^ and control mice at 6 weeks, when AVMs in the retina are most prominent. Evaluation of these organs did not reveal signs of vessel enlargement, hemorrhage, or overall changes in tissue structure (Supplemental Figure 11). We reasoned that this might be due a unique function of pericyte Notch signaling in neural tissue during development. Since angiogenesis has been identified as a likely contributor to AVM formation (41), we decided to explore the effects of loss of pericyte Notch signaling in the embryonic brain vasculature.

The mouse brain undergoes extensive vascular growth and remodeling during embryonic development and may be particularly susceptible to pericyte-specific genetic manipulations (41). Thus, we hypothesized that impaired Notch signaling in embryonic brain pericytes may lead to vascular malformations. Tamoxifen was administered to the pregnant dams at E9.5, E10.5, and E11.5, the stages of vascularization of the CNS during embryonic development (42). The brain vasculature of Rbpj^iΔPC^ and control mice was analyzed at E18.5 (Supplemental Figure 12A). Coronal brain sections of control and Rbpj^iΔPC^ mice were immunostained with IB4 to label the blood vessels and anti-Desmin to identify the pericytes (Supplemental Figure 12B). Consistent with our observations in the developmental retina, we found that vessel diameter was markedly increased in the brain of Rbpj^iΔPC^ compared with control mice. Similarly, we also observed a significant reduction in pericyte coverage in brain sections from mutant mice compared with controls (Supplemental Figure 12C).

These results show that pericyte-Notch signaling is involved in regulating vascular morphogenesis in organs other than the retina, and they suggest a role for pericyte-Notch signaling in preventing vascular malformations in the brain.

### Increased apoptosis in pericytes with deficient Notch signaling.

Our findings show a reduced number of pericytes in mice with perivascular loss of Notch signaling. Notch has also been previously shown to be involved in regulating pericyte proliferation in zebrafish (21). Therefore, to determine if the observed loss of pericytes in the angiogenic front plexus of Rbpj^iΔPC^ mice at P5 was due to a failure of pericytes to expand, we performed an EdU proliferation assay (Supplemental Figure 13A). We found that, at P5, pericytes proliferation was unaffected in retinas from Rbpj^iΔPC^ mice at both the mature capillary plexus and the immature angiogenic front plexus when compared with the control (Supplemental Figure 13B). When pericyte proliferation was assessed at P10, we observed a significant decrease in retinas from Rbpj^iΔPC^ compared with control mice (Supplemental Figure 13C).

**Figure 6:**
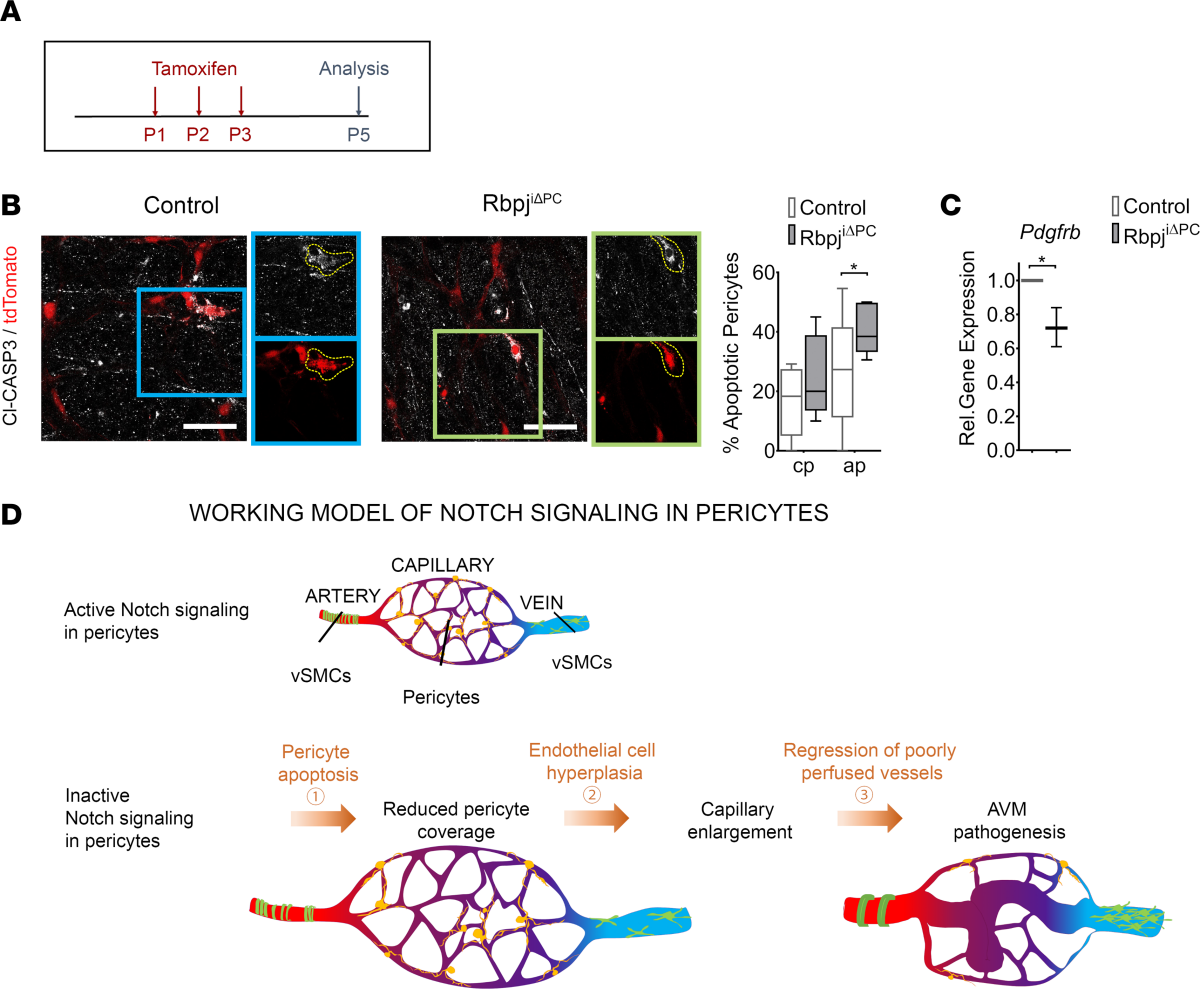
Increased pericyte apoptosis following perivascular inhibition of Notch signaling. Control and Rbpj^iΔPC^ mice bred with Ai9 (TdTomato) reporter mice. (**A**) Offspring were treated with tamoxifen perinatally and analyzed for apoptosis at P5. (**B**) tdTomato reporter (red) utilized to represent pericytes in control and Rbpj^iΔPC^ vasculature. Anti–cleaved Caspase 3 (Cl-CASP3, white) labels apoptotic cells. Boxed regions show higher magnification of apoptotic pericytes. Quantification of pericyte apoptosis in capillary plexus (cp) and apoptotic pericytes (ap) displayed on the right (*n* = 5). (**C**) Gene expression of *Pdgfrb* in FAC-sorted retinal perivascular cells relative to *β-actin*. Box-and-whisker plots show median, minimum, and maximum values. Data were analyzed using unpaired 2-tailed *t* test with Welch’s correction. *P < 0.05. (**D**) Working model of AVM formation in Rbpj^iΔPC^ mice.

Previous work has also shown that loss of vSMCs in *Notch3*-null mice is associated with increased apoptosis in this population (17). However, in these studies, apoptosis in pericytes from *Notch3*-null mice was either not detected (17) or not specifically addressed (16). To investigate if the observed loss of pericytes in Rbpj^iΔPC^ mice was due to increased apoptosis, we immunostained retinas from Rbpj^iΔPC^ and control mice at P5 with anti–cleaved Caspase-3 to detect apoptotic cells. In these experiments, Rbpj^iΔPC^ and control mice were crossed with the Rosa-tdTomato reporter mouse line to allow for easier visualization of this population. Analysis of the samples revealed no significant differences in the capillary plexus but increased levels of apoptosis in pericytes in the angiogenic plexus from Rbpj^iΔPC^ when compared with control mice (Figure 6, A and B).

Taken together, our data point to a critical role for Notch signaling in promoting pericyte survival and proliferation.

### PDGFRβ is downregulated in pericytes with loss of Notch signaling.

Signaling through PDGFRβ is essential for pericyte proliferation and survival (43, 44), and Notch signaling has been reported to regulate the levels of PDGFRβ (14, 20); therefore, we hypothesized that the loss of pericyte survival and proliferation observed in Rbpj^iΔPC^ mutant mice was a result of reduced levels of PDGFRβ. We evaluated the level of expression of *Pdgfrb* in pericytes isolated from retinas of Rbpj^iΔPC^ and control mice at P6. Our results show a significant reduction in *Pdgfrb* levels of pericytes from mutant compared with control mice (Figure 6C).

These data suggest that Notch regulates pericyte survival and proliferation through PDGFRβ.

## Discussion

AVMs are high-flow lesions connecting arteries and veins without an intervening capillary bed. Evidence from multiple studies has reported a lack of pericyte coverage in these lesions in both human patients (9) and in animal models of the disease (45–48). However, whether the lack of pericytes is a cause or a consequence of AVMs and which signaling pathways mediate pericyte loss remains an open question to date. Our findings show how the deficiency of Notch signaling in pericytes results in increased pericyte apoptosis and decrease in pericyte coverage of the vasculature, ultimately leading to the formation of AVMs (Figure 6D). In our model, AVMs seem to form as a consequence of improper vessel remodeling of an immature vascular network. This work sheds light into a potentially novel mechanism of AVM formation as a consequence of poor perivascular coverage and points to the Notch signaling pathway as a critical mediator in this process.

Our observations suggest that AVMs in Rbpj^iΔPC^ mice develop due to the lack of appropriate vessel remodeling. It is widely established that pericytes are critical for vascular maturation (2). Vessel diameter of pericyte-deficient vascular beds is abnormally enlarged, indicative of a role for pericytes in regulating capillary caliber (5, 28, 44). Our findings support a model where enlarged capillary diameter, as a result of pericyte loss in Rbpj^iΔPC^ mice, would provide a path of blood flow with the least vessel resistance, selecting the growth of the wider connections at the expense of the neighboring branches. Over time, this selection of wider connections would give rise to abnormal arteriovenous connections resembling AVMs. Consistent with this model, we observe a reduction in the number of branching points at P10, P14, and 6 weeks and a significant increase in the number of empty collagen sleeves, together with poorly perfused branches at 6 weeks. These findings are characteristic of vessel regression of poorly perfused branches due to flow “steal” from AVMs. In agreement with our interpretation, similar hemodynamic changes have been described to contribute to AVM pathogenesis in other models of the disease (49, 50).

Our data show a reduction in pericyte coverage at P5; however, we did not observe any differences in vessel branching, tip cells, vessel diameter, or EC proliferation at this time point. In contrast, the work from Eilken et al. shows that acute pericyte depletion in the postnatal retina using a genetic model expressing the diphtheria toxin receptor in pericytes, combined with postnatal injections of the toxin, resulted in severe changes in EC angiogenic behavior (27). These differences may be explained by the varying degree of pericyte reduction present in each model. At P6, Eilken et al. report a 60% reduction of pericyte coverage in the angiogenic front, while at P5, the pericyte coverage reduction in the angiogenic front of Rbpj^iΔPC^ mice is 34%. We cannot rule out that, in our model, a small reduction in pericyte coverage at P5 is not enough to render differences in angiogenesis at this early stage, but as pericyte loss becomes more severe, it might have some impact in the angiogenic response at later time points.

Together with a reduction of pericyte coverage, we observed a significant increase in pericyte apoptosis at P5. These findings point to an important role for Notch in pericyte survival. Work from Arboleda-Velasquez et al. describes a role for Notch signaling in protecting pericytes in culture from light-induced cell death (19). Similarly, previous studies described increased apoptosis of vSMCs in *Notch3*-null mice, also suggesting a role for Notch signaling in vSMC survival (17). However, *Notch3*-null mice did not present any changes on pericyte coverage or pericyte apoptosis. It is possible that other Notch receptors expressed in pericytes, such as Notch1, can function to promote pericyte survival in the absence of Notch3 in the *Notch3*-null mice. The exact mechanism on how Notch promotes pericyte survival is not clear, to date. Our data show that retinal pericytes from Rbpj^iΔPC^ mice have reduced *Pdgfrb* expression. Notch signaling has been previously reported to regulate PDGFRβ levels in pericytes in vitro (14, 20); moreover, deactivation of PDGFRβ in retinal pericytes has been reported to cause apoptosis (51). Taken together, our data indicate that Notch signaling mediates pericyte survival through regulation of PDGFRβ levels.

We cannot exclude that Notch has additional functions in pericytes, such as regulating genes or other pathways critical for pericyte migration or association with the endothelium. In ECs, Notch cooperates with Smad4 to regulate N-cadherin. Loss of Notch signaling in ECs results in the loss of N-cadherin (a critical molecular mediating pericyte-EC interactions) and subsequent reduction of perivascular coverage (52). Studies using pericytes in vitro have reported that Notch also regulates N-cadherin in this cell type (20); however, whether this regulation results in loss of pericyte coverage in vivo remains to be established.

Irregular increased expression of α-SMA was detected on the veins of Rbpj^iΔPC^ mice beginning at P10, coinciding with dysregulated vascular maturation and AVM pathogenesis at P10, P14, and 6 weeks. High levels of α-SMA expression are usually associated with arterial vSMCs, consistent with their regulation of blood flow and pressure (13). However, the observed α-SMA^+^ population in the veins of Rbpj^iΔPC^ mice lacked the concentric, ring-like morphology arterial vSMCs exhibit. Rather, these mural cells appear as stellate shaped, indicative of venous vSMCs (3). Veins are abundantly covered by stellate-shaped vSMCs; however, the α-SMA expression in these vessels is commonly weaker than in their arterial counterparts. The most likely explanation for this increase in α-SMA expression in the venous vSMCs is the presence of abnormal hemodynamics and increased blood pressure due to development of arteriovenous shunts. Our findings are consistent with the histology of AVM samples from patients who show hypertensive veins with hypertrophy of the muscular layers (53).

Here, we show that embryonic deficiency of *Rbpj* in pericytes leads to vascular malformations in the embryonic forebrain. These malformations are associated with the loss of pericytes and pronounced capillary enlargement resembling the AVMs observed in Rbpj^iΔPC^ retinas, suggesting that inactivation of Notch signaling in pericytes leads to vascular malformations during vascular development.

Consistent with a role for Notch signaling in pericytes during development, we did not observe abnormal vascular shunting and/or tortuous vessels in the brain, heart, liver, and lung at 6 weeks after postnatal tamoxifen treatment. However, our evaluation using H&E sections might have missed the presence of small AVMs and/or subtle pericyte abnormalities in these organs.

Our work indicates that AVM pathogenesis in Rbpj^iΔPC^ mice arises from deficient canonical Notch signaling pathway in pericytes. We show that overexpression of dnMAML in pericytes and loss of Notch1 and Notch3 in pericytes both lead to enlarged, tortuous connections between arteries and veins, recapitulating pericyte loss and AVM phenotypes of Rbpj^iΔPC^ mice. In contrast with our findings, a recent study suggests that loss of Rbpj in pericytes elicits vascular malformations in the brain, independently of Notch signaling (15). We did not observe postnatal vascular malformations in the brain of our mutant mice, possibly due to differences in the PDGFRβ-CreERT^2^ mouse strains used or the background of the different mouse models. Another possible explanation for the observed discrepancies might lay in the microbiome of the different mice and/or animal facilities, since the microbiome has been reported to play an important role in the development of cerebral vascular malformations (54).

In this manuscript, we show how deficient Notch in pericytes results in AVMs. There are several studies evaluating pericytes in AVMs. The work of Winkler et al. shows that human brain AVMs present reduced pericyte coverage (9). Moreover, AVMs from patients with prior AVM rupture presented with the lowest pericyte coverage (9). Additional evidence for the relevance of pericytes in AVM pathogenesis comes from mouse models of hereditary hemorrhagic telangiectasia (HHT), a rare genetic disease that develops AVMs as a hallmark of its pathology. Retinal and brain lesions in HHT mouse models also exhibit a reduction of pericyte coverage associated with the development of AVMs (45, 46, 48, 55). However, to our knowledge, our study is the first to provide a causative link between pericyte loss and AVM development in mice.

Today, AVMs are mostly treated by crude and high-risk techniques, including endovascular obliteration and surgical resection (1). There is a clear need for noninvasive therapies targeting biologic mechanisms of lesion genesis, maintenance, and potential regression. Previous efforts using thalidomide have demonstrated that strategies promoting the stability and maturation of the vascular wall by targeting pericyte-EC communication can have beneficial effects on bleeding by normalizing AVM lesion (47, 55). Pericytes are emerging therapeutic targets through pharmacological manipulations of Notch signaling. We propose that strategies focus on enhancing Notch signaling in perivascular cells; for example, Notch3 activating antibodies, which have already been shown to prevent mural cell loss in CADSAIL mouse models (56), might provide a new, noninvasive avenue to treat AVMs.

## Methods

### Mice.

*PDGFRβ-P2A-CreER^T2^* (*Pdgfrb^tm1.1[cre/ERT2]Csln^*) were generated in our group as previously described (22), *Rbpj^flox^* (23) were obtained from Tasuki Honji (Kyoto University, Kyoto, Japan), *Tg(Acta2-cre/ERT2)12Pcn* (*Acta2-CreER^T2^*) (31) were obtained from Pierre Chambon (Institute for Genetics and Cellular and Molecular Biology/University of Strasbourg Institute for Advanced Study, Strasbourg, France), and *Gt(ROSA)26Sor^tm1(MAML)Wsp^* (*dnMAML^flox^*) (37) were obtained from Warren Pear (University of Pennsylvania, Philadelphia, Pennsylvania, USA). *Gt(ROSA)26Sor^tm1(cre/ERT2)/Tyj^/J* (*ROSA26-CreER^T2^*) (39), *Notch3^tm1Grid^*/2J (*Notch3*-null) (40), *Notch1^tm2Rko^*/GridJ (*Notch1^flox^*) (38), *Tg(Cp-HIST1H2BB/Venus)47Hadj* (*CBF:H2B-Venus*) (32), *Gt(ROSA)26Sor^tm9(CAG–tdTomato)Hze^* (Ai9 mice) (24), and C57BL/6J mice were obtained from Jackson Laboratory.

*Notch3^flox^* mice were a gift of Jan Kitajewski (University of Illinois at Chicago, Chicago, Illinois). They were generated using the RP24-186F13 BAC clone (purchased from BACPAC resource) and transformed into SW106 recombineering bacterial strain. A loxP site (L83) was inserted upstream of the promoter, and a Frt-Neo-Frt-LoxP (FNFL) cassette was inserted in the intron downstream of Exon1. A DNA fragment containing 2 kb upstream of the first LoxP site, the floxed promoter and exon1, the FNFL cassette, and the 5 kb downstream of the FNFL was retrieved into a plasmid (pMCS-DTA) to complete the *Notch3^flox^* vector, pMCS-Notch3-DTA. This targeting vector was linearized and electroporated into KV1 (129B6 hybrid) ES cells, and targeted ES clones were injected into C57BL/6N blastocysts to generate male chimeras, which were bred to B6.Cg-Tg(ACTFLPe)9205Dym/J females (from Jackson Laboratory) to remove the neo cassette.

For all postnatal studies, 250 μg/g of tamoxifen (MilliporeSigma) in corn oil (MilliporeSigma) was administered by oral gavage to nursing moms at P1, P2, and P3. For embryonic studies, 3 mg/day of tamoxifen was administered by oral gavage to the pregnant dam daily from E9.5–E11.5.

All mice were maintained in a pure C57BL/6J background. Females and males were used indistinctively in these studies.

### Retina isolation and immunofluorescence.

Eyes were enucleated following mouse sacrifice at P5, P10, P14, and 6 weeks. Collected eyes were fixed in 4% formaldehyde (Thermo Fisher Scientific) for 1 hour at 4°C on a nutator. Following fixation, eyes were washed with ice-cold 1× PBS solution. Retinas were dissected and permeabilized in 1× PBS containing 1% BSA (Fisher Bioreagents) and 0.5% Triton X-100 (Fisher Bioreagents) overnight at 4°C on a nutator. Samples were then immunostained in PBLEC (5% Triton X-100, 1M MgCl_2_, 1M CaCl_2_, and 1M MnCl_2_ in 1× PBS) overnight at 4°C with Biotinylated IB4 (1:50; Vector Laboratories, B-1205) and/or the following antibodies: anti-CD31 (1:100; BD Pharmingen, 553370), anti–α-SMA-FITC (1:200; MilliporeSigma, F3777), anti–α-SMA-Cy3 (1:200; MilliporeSigma, C6198), anti-NG2 (1:700; MilliporeSigma, AB5320), anti-NG2 (1:700; R&D Systems, MAB6689), anti-Desmin (1:500; Abcam, AB15200), anti–Collagen IV (1:500; Cosmo Bio, LSL-LB-0445), anti–Cleaved Caspase-3 (1:400; Cell Signaling Technology, 9964), anti-Erg1 (1:500; Abcam AB92513), and anti–VE Cadherin (1:200; BD Pharmingen, 555289) in the permeabilizing solution described above. Following primary antibody staining, retinas were washed and incubated with Alexa Fluor–conjugated secondary antibodies (1:700; Invitrogen). Immunostained retinas were postfixed with 4% formaldehyde and flat-mounted in Vectashield (Vector Laboratories). Whole-mount retina images were acquired using Zeiss Axio Zoom.V16 microscope. Confocal stacked images were captured using Zeiss LSM 880 confocal microscope. All images were analyzed using ImageJ (NIH).

### FACS of retinal pericytes.

Retinas from P6 pups expressing the Ai9 reporter were collected in ice-cold DMEM (Thermo Fisher Scientific, 11885-084). Minced samples were transferred to a digestion solution of 0.07% Collagenase I (Thermo Fisher Scientific, 17100-017) in DMEM and digested for 7 minutes at 37°C. Following digestion, tissues were mechanically disrupted using an 18 G needle. Resulting tissue homogenates were filtered through a 100 μM cell strainer (Falcon) and centrifuged at 400*g* for 5 minutes. The cell pellet was resuspended in 300 μL of DMEM with DAPI (1 μM) to exclude dead cells. Cells were sorted directly into ice-cold DMEM using the MoFlo Astrios EQ Cell Sorter (Beckman Coulter). Each experimental sample was derived from littermate control and Rbpj^iΔPC^ mice (pooled retinas).

### RNA isolation, cDNA synthesis, and qPCR.

RNA from sorted pericytes was isolated using PicoPure RNA Isolation Kit (Arcturus, KIT0202) according to the manufacturer’s instructions. cDNA was generated using the Verso cDNA Synthesis Kit (Thermo Fisher Scientific, AB-1453). Quantitative PCR (qPCR) was performed on ABI ViiA7 (Invitrogen) using the Fast SYBR Green Master Mix (Applied Biosystems), and the following forward and reverse primer pairs for *Pdgfrb*: forward, 5′ - TTCCAGGAGTGATACCAGCTT - 3′ ; reverse, 5′ - AGGGGGCGTGATGACTAGG - 3′. As indicated in figure legends, normalized transcript levels are relative to the levels of *β-actin*. For *Rbpj* and *Gapdh*, qPCR was performed using the TaqMan Gene Expression Master Mix (Applied Biosystems), and the following TaqMan gene expression probes (from Thermo Fisher Scientific): *Rbpj* (Mm03053645_s1) and *Gapdh* (Mm99999915_g1) as the housekeeping gene. All relative gene expression analyses were performed using the comparative C_t_ method with triplicate reactions for each sample evaluated.

### In vivo proliferation assay.

Pups were injected i.p. with 30 mg/kg of 5-ethyl-2-deoxyuridine (EdU) solution (Invitrogen, A10044) 4 hours before collection as previously described (27). Retinas were dissected and stained as described above. Following secondary antibody staining, EdU labeling was detected using Click-it-EdU Alexa Fluor-A488 Imaging Kit (Invitrogen, C10337).

### Systemic vessel casting perfusion.

As previously described by Park et al. (57), systemic vascular casting was performed by injecting blue latex compound (Connecticut Valley Biological Supply Company) or microfil (Flow Tech Inc.) into the left ventricle of mice (32). Eyes were enucleated and fixed overnight in 4% formaldehyde at 4°C. Retinas were dissected and permeabilized as described above. They were next incubated overnight at 4°C with IB4 (1:50; Vector Laboratories, B-1205). The retinas were then incubated in a working solution of DAB peroxidase substrate (Vector Laboratories). After application, they were washed using 1× PBS and flat-mounted in Vectashield (Vector Laboratories). Tiled images were acquired using Zeiss Axio Imager 2 inverted microscope.

### Lectin perfusion.

Injections of biotinylated Tomato Lectin (Vector Laboratories, B-1175) were used to label perfused blood vessels as previously described (58). A total of 50 μg of Tomato Lectin (2 mg/mL, prepared in DPBS) was injected into the inferior vena cava. Eyes were enucleated and fixed in 4% formaldehyde for 1 hour and followed by retinal dissection, permeabilization, and staining as described above.

### Western blotting.

Pooled retinas (2–3 mice) were lysed with 250 μL of RIPA buffer (Cell Signaling Technology, 9806) containing 1× protease inhibitor (Thermo Fisher Scientific, 78430), 1× phosphatase inhibitor (Thermo Fisher Scientific, 78420), and 1 mM of DDT. Using a Bullet Blender (MIDSCI, BBX24B), retinas were lysed at speed 8 for 30 seconds in 1.5 mL RINO screw-cap tubes prefilled with stainless steel beads. Western blot was performed using anti-Notch3 (1:1000; Santa Cruz Biotechnology Inc., sc-5593) and anti-Gapdh (1:30,000; Proteintech, 10494-1-AP) primary antibodies in blocking solution (2% milk, 2% BSA, and 1× TBST 0.1% Tween 20). HRP-conjugated secondary antibody (Cell Signaling Technology, 70745) were used for detection.

### Histology.

Tissues were fixed immediately after collection in 100% neutral buffered formalin at room temperature for 24–48 hours. After fixation, tissues were placed in 70% ethanol. Samples were paraffin embedded and sectioned at 5 μm. Embedding, sectioning, and H&E staining were performed by University of Illinois at Chicago’s Research Histology and Tissue Imaging Core.

### Brain isolation and immunofluorescence.

Embryo brains were isolated and fixed in 4% formaldehyde overnight at 4°C. After fixation, they were transferred to 30% sucrose solution and embedded in OCT compound (Tissue Tek), frozen, and stored in –80°C. Coronal sections (40 μm thick) were stained following retinal immunostaining protocol as described above. Immunostained brain sections were postfixed with 4% formaldehyde and flat-mounted in Vectashield (Vector Laboratories). Confocal stacked images were captured using Zeiss LSM 880 confocal microscope. All images were analyzed using ImageJ (NIH).

### Image analysis.

All confocal images were acquired using the ZEISS LSM 880 with Airyscan for quantification and analysis. Vessel area was quantified as threshold value of EC area per total field area. Branching points were defined as intersections between vessels. The total number of branching points per area (mm^2^) was calculated. Five measurements of vessel width were taken of each artery and vein, and 10 measurements of vessel width of capillary fields were acquired; the average width was plotted. The number of tip cells in the angiogenic front were counted in each field (mm). Growth of vessels was measured from the center of the retina to the tip of the peripheral vessels. Retinal vasculature staining with EC marker and pericyte marker was performed. Pericyte area was quantified as a threshold value of pericyte marker immunofluorescence. To quantify pericyte coverage, the percentage of pericyte area per ECs area was recorded. Retinal vasculature staining with EC marker and vSMC marker was performed. vSMC area was quantified as a threshold value of α-SMA immunofluorescence. Quantification of vSMC coverage was calculated by normalizing α-SMA staining to EC area. Retinas were labeled for EdU and endothelial nuclei marker (Erg1) for EC proliferation. The number of Erg1^+^ cells were counted. The ratio of total number of EdU^+^/Erg1^+^ cells was calculated. For pericyte proliferation, pericytes were labeled using the tdTomato (Ai9) reporter expression. The number of tdTomato^+^ cells were counted, and the ratio of EdU^+^/tdTomato^+^ cells was calculated. tdTomato recombination was measured as percentage of tdTomato reporter expression associated with α-SMA^+^ vSMCs on arteries. Venus expression was quantified as the number of Venus^+^ cells per α-SMA^+^ area on arteries. Using VE-Cadherin membrane staining, 40 capillary EC sizes were measured per capillary fields, and the average size was plotted. Retinal vasculature staining with EC marker and vascular basement membrane marker (Collagen IV) was performed. The number of Collagen IV^+^/IB4^+^ sleeves was counted in the capillary vasculature. The ratio of total number of Collagen IV^+^/IB4^–^ sprouts was calculated per area (mm^2^). Retinal vasculature staining with EC marker and for perfused Tomato Lectin was performed. The number of Lectin^+^/CD31^+^ vessels were counted in the capillary vasculature. The ratio of total number of Lectin^–^/CD31^+^ vessels was calculated.

### Statistics.

Data were plotted and analyzed in Prism GraphPad. Comparison between different data sets was made using unpaired 2-tailed *t* test with Welch’s correction and 1-way ANOVA with Tukey’s post hoc test. *P* < 0.05 was considered to be statistically different.

### Study approval.

Animal experiments were performed in compliance with the Animal Care Committee guidelines at the University of Illinois at Chicago under protocol ACC 17-131. All animals were maintained and treated with humane care according to the *Guide for the Care and Use of Laboratory Animals* (National Academies Press, 2011).

## Author contributions

WB and BB conducted experiments, acquired data, and analyzed the data. TN and HC designed the studies, conducted experiments, acquired data, analyzed the data, and wrote the manuscript.

## Supplementary Material

supplemental data

## Version 1 11/05/2020

Electronic publication

## References

[1] Solomon RA, Connolly ES (2017). Arteriovenous Malformations of the Brain. N Engl J Med.

[2] Armulik A, Genové G, Betsholtz C (2011). Pericytes: developmental, physiological, and pathological perspectives, problems, and promises. Dev Cell.

[3] Hartmann DA, Underly RG, Grant RI, Watson AN, Lindner V, Shih AY (2015). Pericyte structure and distribution in the cerebral cortex revealed by high-resolution imaging of transgenic mice. Neurophotonics.

[4] Winkler EA, Bell RD, Zlokovic BV (2011). Central nervous system pericytes in health and disease. Nat Neurosci.

[5] Hellström M (2001). Lack of pericytes leads to endothelial hyperplasia and abnormal vascular morphogenesis. J Cell Biol.

[6] Shin ES, Sorenson CM, Sheibani N (2014). Diabetes and retinal vascular dysfunction. J Ophthalmic Vis Res.

[7] Lendahl U, Nilsson P, Betsholtz C (2019). Emerging links between cerebrovascular and neurodegenerative diseases-a special role for pericytes. EMBO Rep.

[8] Winkler EA, Sagare AP, Zlokovic BV (2014). The pericyte: a forgotten cell type with important implications for Alzheimer’s disease?. Brain Pathol.

[9] Winkler EA (2018). Reductions in brain pericytes are associated with arteriovenous malformation vascular instability. J Neurosurg.

[10] Siebel C, Lendahl U (2017). Notch Signaling in Development, Tissue Homeostasis, and Disease. Physiol Rev.

[11] Kopan R (2012). Notch signaling. Cold Spring Harb Perspect Biol.

[12] Fouillade C, Monet-Leprêtre M, Baron-Menguy C, Joutel A (2012). Notch signalling in smooth muscle cells during development and disease. Cardiovasc Res.

[13] Vanlandewijck M (2018). A molecular atlas of cell types and zonation in the brain vasculature. Nature.

[14] Kofler NM, Cuervo H, Uh MK, Murtomäki A, Kitajewski J (2015). Combined deficiency of Notch1 and Notch3 causes pericyte dysfunction, models CADASIL, and results in arteriovenous malformations. Sci Rep.

[15] Diéguez-Hurtado R (2019). Loss of the transcription factor RBPJ induces disease-promoting properties in brain pericytes. Nat Commun.

[16] Liu H, Zhang W, Kennard S, Caldwell RB, Lilly B (2010). Notch3 is critical for proper angiogenesis and mural cell investment. Circ Res.

[17] Henshall TL (2015). Notch3 is necessary for blood vessel integrity in the central nervous system. Arterioscler Thromb Vasc Biol.

[18] Volz KS (2015). Pericytes are progenitors for coronary artery smooth muscle. Elife.

[19] Arboleda-Velasquez JF, Primo V, Graham M, James A, Manent J, D’Amore PA (2014). Notch signaling functions in retinal pericyte survival. Invest Ophthalmol Vis Sci.

[20] Schulz GB (2015). Cerebral Cavernous Malformation-1 Protein Controls DLL4-Notch3 Signaling Between the Endothelium and Pericytes. Stroke.

[21] Wang Y, Pan L, Moens CB, Appel B (2014). Notch3 establishes brain vascular integrity by regulating pericyte number. Development.

[22] Cuervo H (2017). PDGFRβ-P2A-CreER^T2^ mice: a genetic tool to target pericytes in angiogenesis. Angiogenesis.

[23] Han H (2002). Inducible gene knockout of transcription factor recombination signal binding protein-J reveals its essential role in T versus B lineage decision. Int Immunol.

[24] Madisen L (2010). A robust and high-throughput Cre reporting and characterization system for the whole mouse brain. Nat Neurosci.

[25] Zhang R, Zhu W, Su H (2016). Vascular Integrity in the Pathogenesis of Brain Arteriovenous Malformation. Acta Neurochir Suppl.

[26] Gariano RF, Gardner TW (2005). Retinal angiogenesis in development and disease. Nature.

[27] Eilken HM (2017). Pericytes regulate VEGF-induced endothelial sprouting through VEGFR1. Nat Commun.

[28] Ogura S (2017). Sustained inflammation after pericyte depletion induces irreversible blood-retina barrier breakdown. JCI Insight.

[29] Ivanova E, Corona C, Eleftheriou CG, Bianchimano P, and Sagdullaev BT. Retina-specific targeting of pericytes reveals structural diversity and enables control of capillary blood flow. bioRxiv. 10.1101/2020.05.29.124586 Published May 31, 2020. Accessed September 29, 2020 PMC7867582 32812219

[30] Hellström M, Kalén M, Lindahl P, Abramsson A, Betsholtz C (1999). Role of PDGF-B and PDGFR-beta in recruitment of vascular smooth muscle cells and pericytes during embryonic blood vessel formation in the mouse. Development.

[31] Wendling O, Bornert JM, Chambon P, Metzger D (2009). Efficient temporally-controlled targeted mutagenesis in smooth muscle cells of the adult mouse. Genesis.

[32] Ilagan MX, Lim S, Fulbright M, Piwnica-Worms D, Kopan R (2011). Real-time imaging of notch activation with a luciferase complementation-based reporter. Sci Signal.

[33] Stratman AN, Davis GE (2012). Endothelial cell-pericyte interactions stimulate basement membrane matrix assembly: influence on vascular tube remodeling, maturation, and stabilization. Microsc Microanal.

[34] Stratman AN, Malotte KM, Mahan RD, Davis MJ, Davis GE (2009). Pericyte recruitment during vasculogenic tube assembly stimulates endothelial basement membrane matrix formation. Blood.

[35] Díaz-Trelles R (2016). Notch-independent RBPJ controls angiogenesis in the adult heart. Nat Commun.

[36] Kulic I (2015). Loss of the Notch effector RBPJ promotes tumorigenesis. J Exp Med.

[37] Tu L (2005). Notch signaling is an important regulator of type 2 immunity. J Exp Med.

[38] Yang X, Klein R, Tian X, Cheng HT, Kopan R, Shen J (2004). Notch activation induces apoptosis in neural progenitor cells through a p53-dependent pathway. Dev Biol.

[39] Ventura A (2007). Restoration of p53 function leads to tumour regression in vivo. Nature.

[40] Krebs LT (2003). Characterization of Notch3-deficient mice: normal embryonic development and absence of genetic interactions with a Notch1 mutation. Genesis.

[41] Mullan S, Mojtahedi S, Johnson DL, Macdonald RL (1996). Embryological basis of some aspects of cerebral vascular fistulas and malformations. J Neurosurg.

[42] Tata M, Ruhrberg C, Fantin A (2015). Vascularisation of the central nervous system. Mech Dev.

[43] Sweeney MD, Ayyadurai S, Zlokovic BV (2016). Pericytes of the neurovascular unit: key functions and signaling pathways. Nat Neurosci.

[44] Park DY (2017). Plastic roles of pericytes in the blood-retinal barrier. Nat Commun.

[45] Chen W (2013). Reduced mural cell coverage and impaired vessel integrity after angiogenic stimulation in the Alk1-deficient brain. Arterioscler Thromb Vasc Biol.

[46] Thalgott J, Dos-Santos-Luis D, Lebrin F (2015). Pericytes as targets in hereditary hemorrhagic telangiectasia. Front Genet.

[47] Lebrin F (2010). Thalidomide stimulates vessel maturation and reduces epistaxis in individuals with hereditary hemorrhagic telangiectasia. Nat Med.

[48] Crist AM, Lee AR, Patel NR, Westhoff DE, Meadows SM (2018). Vascular deficiency of Smad4 causes arteriovenous malformations: a mouse model of Hereditary Hemorrhagic Telangiectasia. Angiogenesis.

[49] Corti P (2011). Interaction between alk1 and blood flow in the development of arteriovenous malformations. Development.

[50] Murphy PA (2008). Endothelial Notch4 signaling induces hallmarks of brain arteriovenous malformations in mice. Proc Natl Acad Sci USA.

[51] Geraldes P (2009). Activation of PKC-delta and SHP-1 by hyperglycemia causes vascular cell apoptosis and diabetic retinopathy. Nat Med.

[52] Li F (2011). Endothelial Smad4 maintains cerebrovascular integrity by activating N-cadherin through cooperation with Notch. Dev Cell.

[53] Miller DD, Gupta A (2016). Histopathology of vascular anomalies: update based on the revised 2014 ISSVA classification. Semin Cutan Med Surg.

[54] Tang AT (2017). Endothelial TLR4 and the microbiome drive cerebral cavernous malformations. Nature.

[55] Zhu W (2018). Thalidomide Reduces Hemorrhage of Brain Arteriovenous Malformations in a Mouse Model. Stroke.

[56] Machuca-Parra AI (2017). Therapeutic antibody targeting of Notch3 signaling prevents mural cell loss in CADASIL. J Exp Med.

[57] (2009). Real-time imaging of de novo arteriovenous malformation in a mouse model of hereditary hemorrhagic telangiectasia. J Clin Invest.

[58] Cuervo H, Nielsen CM, Simonetto DA, Ferrell L, Shah VH, Wang RA (2016). Endothelial notch signaling is essential to prevent hepatic vascular malformations in mice. Hepatology.

