# Supplementary material for: Deficiency of Notch signaling in pericytes results in arteriovenous malformations
Source: JCI Insight. 2020 Nov 5;5(21):e125940. doi: 10.1172/jci.insight.125940 (PMC7710269; doi:10.1172/jci.insight.125940)
Supplement: supplemental data [file jciinsight-5-125940-s140.pdf]

SUPPLEMENTAL FIGURE 1

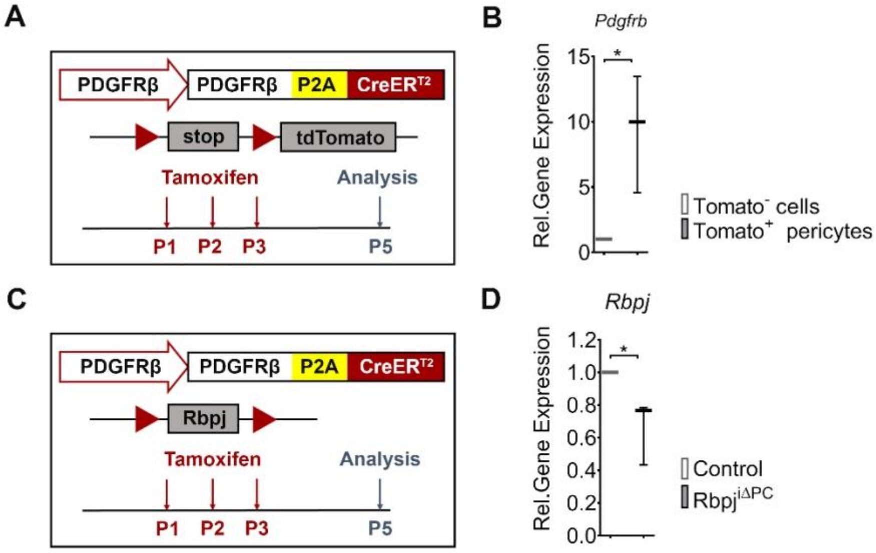

Supplemental Figure 1. Efficiency of tamoxifen-inducible deletion of *Rbpj* in retinal pericytes. Retinas from Rbpj$^{i\Delta PC}$ and control mice were enzymatically digested; single cell suspension was FACs sorted and later evaluated for gene expression. (A) Diagram of tamoxifen administration to *PDGFRβ-P2A-CreRT*^{T2} mice bred with the Ai9 (tdTomato) reporter mice and retinal harvest at P6. (B) Gene expression fold change of *Pdgfrb* in FACs-sorted retinal perivascular cells relative to *β-actin*. (C) Diagram of tamoxifen administration to *PDGFRβ-P2A-CreRT*^{T2} mice bred with *Rbpj*^{flox} and reporter mice and retinal harvest at P6. (D) Gene expression fold change of *Rbpj* in tdTomato$^+$ FACs-sorted retinal perivascular cells relative to *Gapdh*. n = 3 independent experiments using pooled retinas from 3-5 mice per experiment. Box-and-whisker plots show median, minimum and maximum values. Data analyzed using unpaired two-tailed t-test. * $p < 0.05$.

SUPPLEMENTAL FIGURE 2

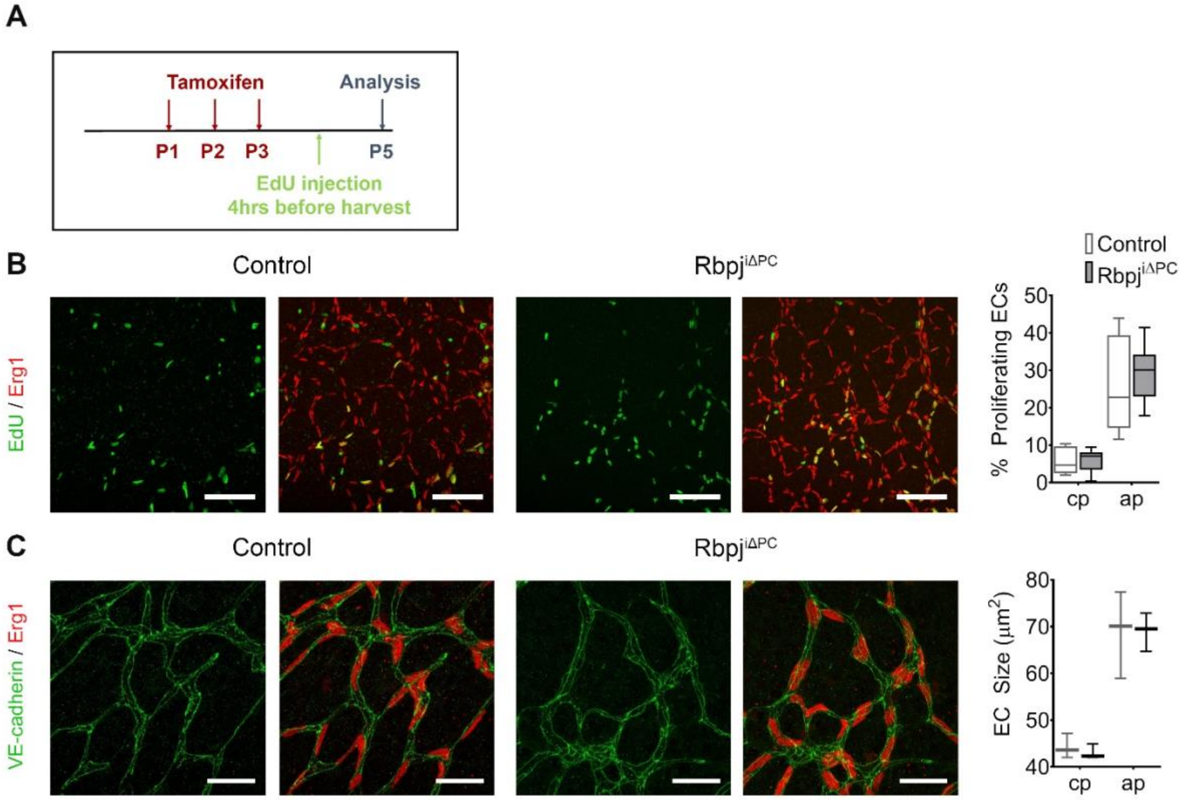

Supplemental Figure 2. Loss of perivascular Notch signaling does not influence endothelial cell expansion during early angiogenesis. (A) Diagram of tamoxifen and EdU administration to postnatal control and Rbpj$^{i\Delta PC}$ mice and analysis at P5. (B) Representative images of the capillary plexus and quantification of retinal vasculature of control and Rbpj$^{i\Delta PC}$ mice stained for EdU (green) and Early growth response factor 1 (Erg1, red) to label proliferating endothelial cells (ECs). Scale bars, 100 µm. Quantification of % proliferating ECs in control and Rbpj$^{i\Delta PC}$ mice (n = 5-7). (C) Higher magnification images of the capillary plexus and quantification of ECs stained for Erg1 (red) and membrane marker vascular endothelial cadherin (VE-Cadherin, green) to evaluate EC size in control and Rbpj$^{i\Delta PC}$ mice. Scale bars, 30 µm. Quantification of EC in control and Rbpj$^{i\Delta PC}$ mice (n = 3). Data was analyzed using unpaired two-tailed test with Welch's correction.

SUPPLEMENTAL FIGURE 3

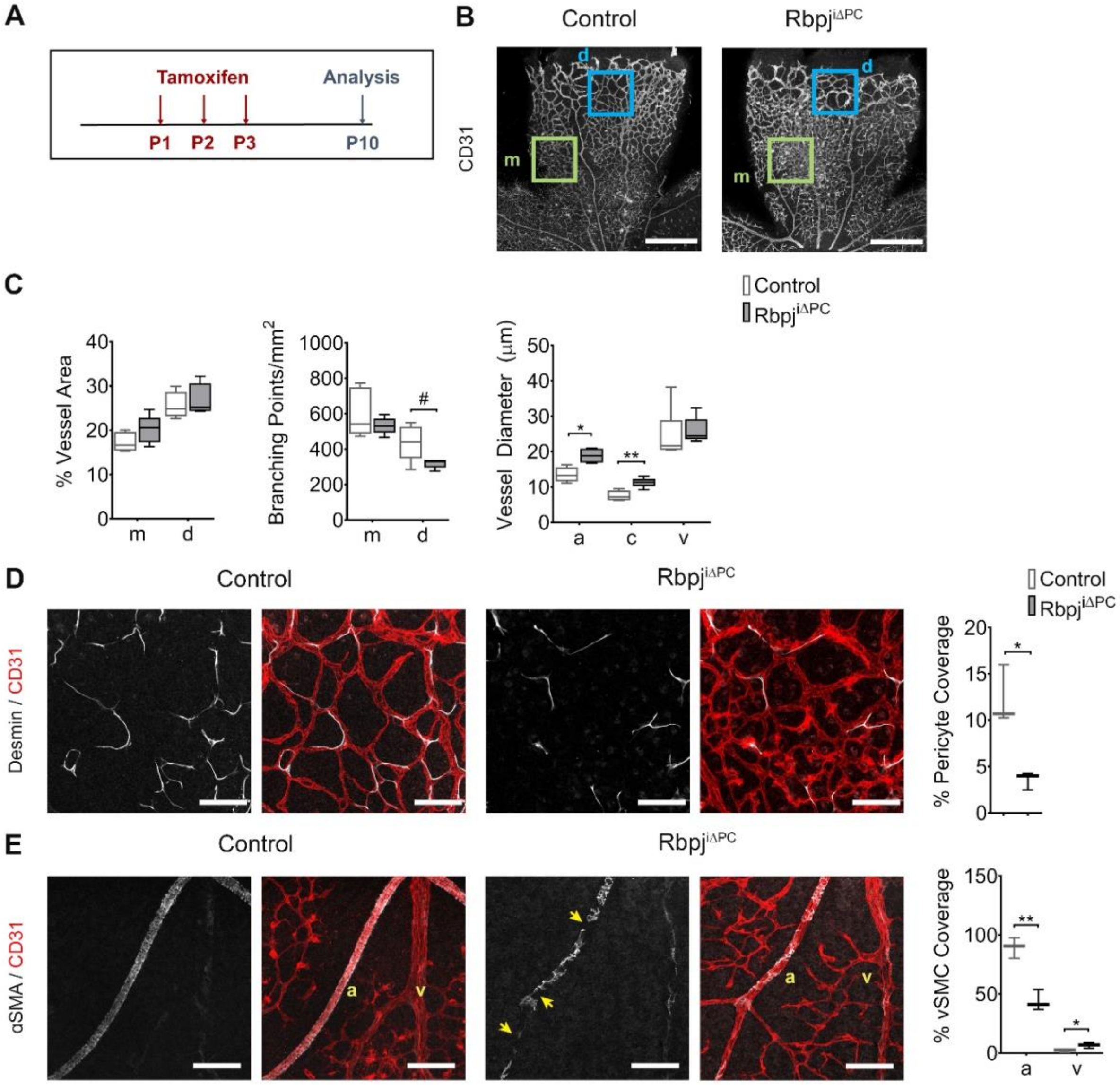

Supplemental Figure 3. Impaired vessel remodeling following inhibition of perivascular Notch signaling. (A) Diagram of tamoxifen administration to postnatal control and Rbpj$^{i\Delta PC}$ mice and analysis at P10. (B) Confocal images showing P10 retinal vasculature stained with anti-CD31 (white). Blue box represents the distal capillary plexus (d) and green box represents the middle capillary plexus (m) utilized for analysis. Scale bars, 400 µm (C) Quantification of % vessel area (n = 4-5), branching points/mm^2 (n = 5) and vessel diameter (n = 4-6) in control and Rbpj$^{i\Delta PC}$ mice. (D) High-magnification confocal images of anti-Desmin (white) and anti-CD31 (red) staining of pericytes and the capillaries respectively. Scale bars, 100 µm. Quantification of % pericyte coverage (n = 3) in control and Rbpj$^{i\Delta PC}$ mice. (F) CD31 (red) highlights the arteries and veins and alpha-smooth muscle actin (αSMA) (white) represents vascular smooth muscle cells (vSMCs). Note CD31$^+$ arteries have reduced αSMA$^+$ vSMCs, as indicated by arrows. Scale bars, 100 µm. Quantification of % vSMC coverage (n = 3) in control and Rbpj$^{i\Delta PC}$ mice. Box-and-whisker plots show median, and minimum and maximum values. a = artery, c = capillary and v = vein. Data analyzed using unpaired two-tailed t-test with Welch's correction. * $p < 0.05$, ** $p < 0.01$, # $p = 0.0582$.

SUPPLEMENTAL FIGURE 4

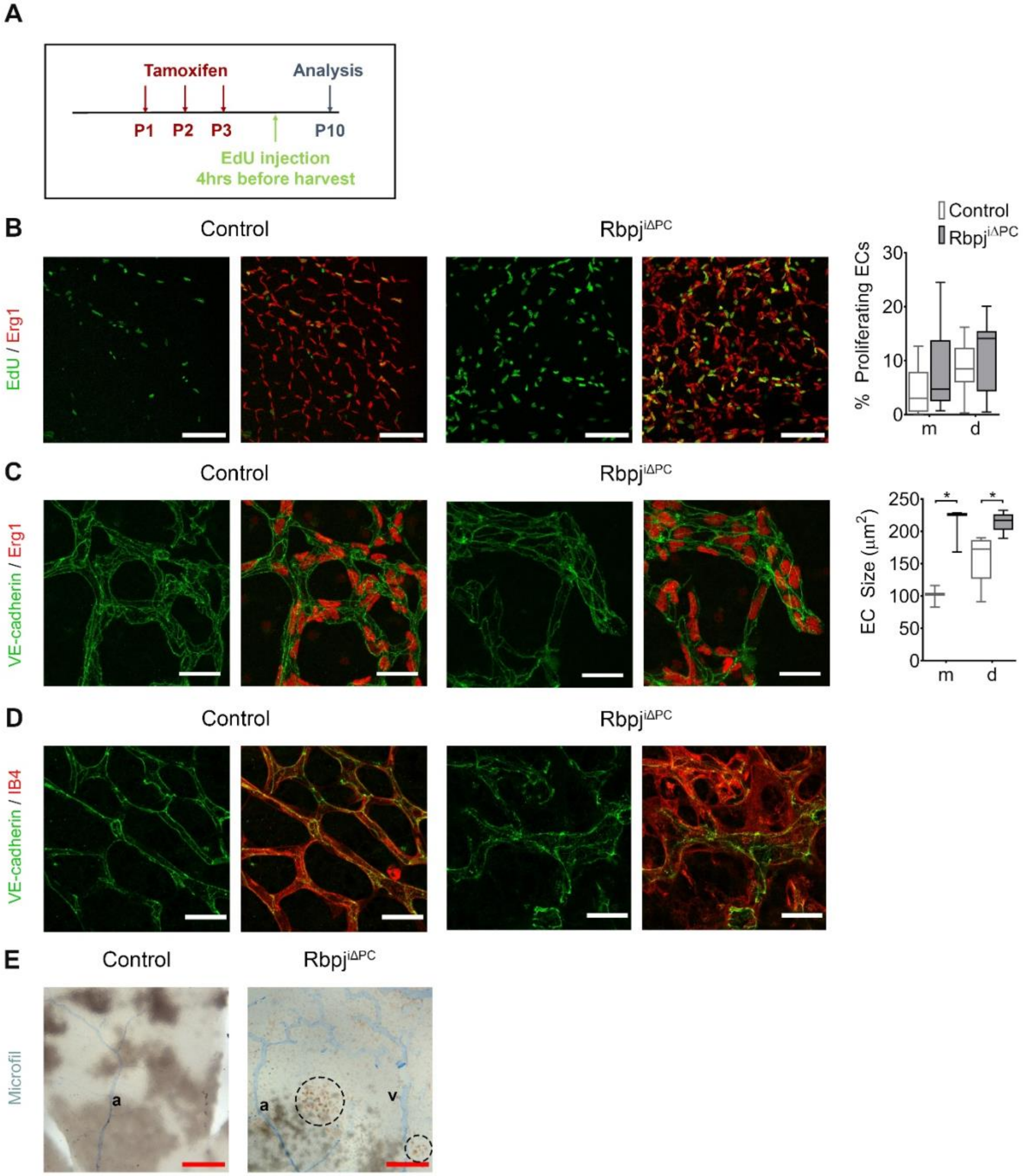

Supplemental Figure 4. Loss of perivascular Notch signaling results in increased endothelial cell size during vessel remodeling. (A) Diagram of tamoxifen and EdU administration to postnatal control and Rbpj$^{i\Delta PC}$ mice and analysis at P10. (B) Representative images of middle capillaries and quantification of retinal vasculature of control and Rbpj$^{i\Delta PC}$ mice stained for EdU (green) and Early growth response factor 1 (Erg1, red) to label proliferating endothelial cells (ECs). Scale bars, 100 µm. Quantification of % proliferating ECs in control and Rbpj$^{i\Delta PC}$ mice (n = 7-9). (C) High magnification images of distal capillaries and quantification of ECs stained for Erg1 (red) and membrane marker vascular endothelial cadherin (VE-Cadherin, green) to evaluate EC size in control and Rbpj$^{i\Delta PC}$ mice. Scale bars, 30 µm. Quantification of ECs in control and Rbpj$^{i\Delta PC}$ mice (n = 3-5). (D) High magnification images of middle capillaries stained for endothelial cell maker Isolectin B4 (IB4, red) and VE-cadherin (green) demonstrate discontinuous endothelial cell-cell junctions Scale bars, 30 µm. (E) Representative brightfield images of microfilm perfused retinal vessels of control and Rbpj$^{i\Delta PC}$ mice. Dashed black circles highlight hemorrhage in Rbpj$^{i\Delta PC}$ retinas. Scale bars, 260 µm. Data was analyzed using unpaired two-tailed test with Welch's correction. * $p < 0.05$

SUPPLEMENTAL FIGURE 5

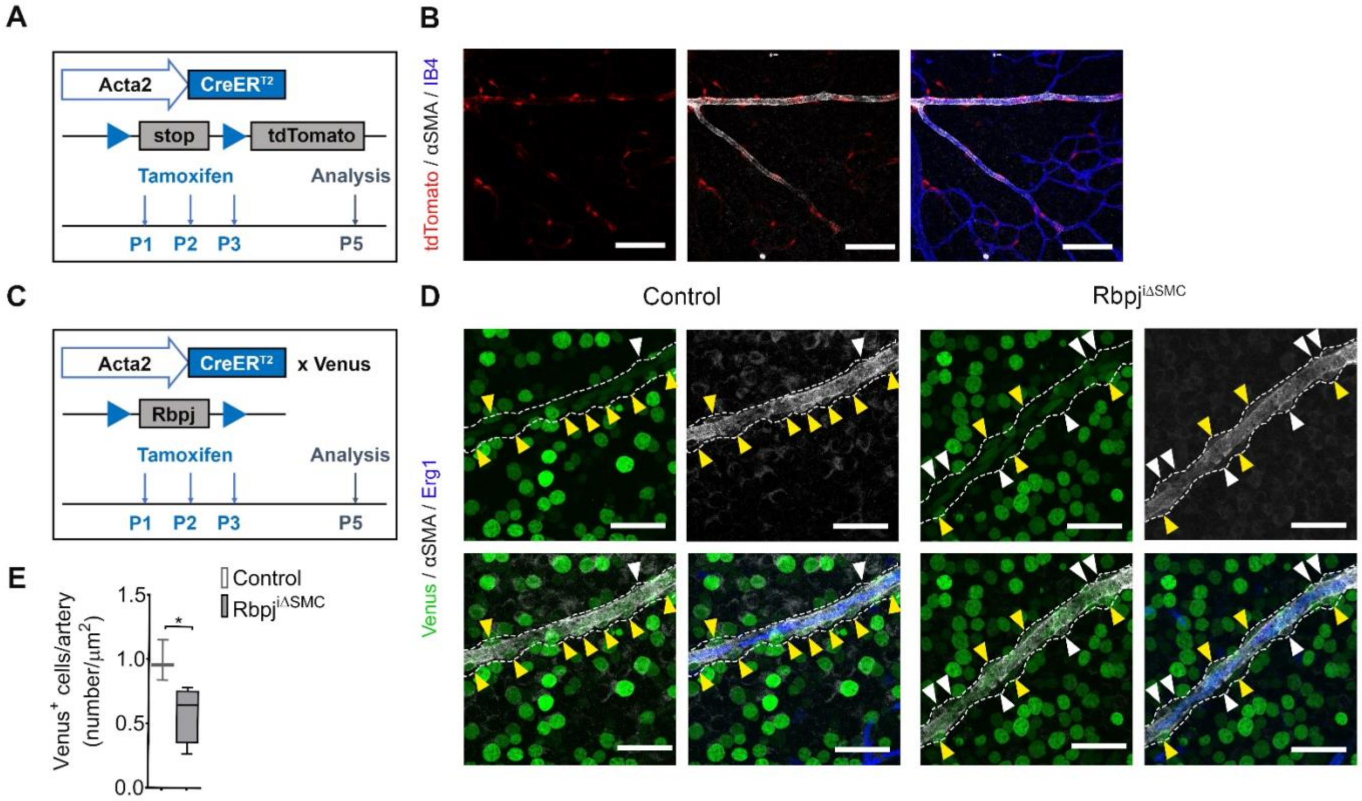

Supplemental Figure 5. Efficiency of tamoxifen-inducible deletion of *Rbpj* in retinal vascular smooth muscle cells. (A) Diagram of tamoxifen administration to *Acta2-CreRT^{T2}* mice bred with the Ai9 (tdTomato) reporter mice and retinal harvest at P5 for analysis. (B) Retinal vasculature stained for alpha-smooth muscle actin (αSMA, white) and Isolectin B4 (IB4, blue) to label vascular smooth muscle cells (vSMCs) and the endothelium, respectively. Expression of the tdTomato reporter is in red. Scale bars, 100 µm. (C) Diagram of tamoxifen administration to *Acta2-CreRT^{T2}* mice bred with *Rbpj^{flox}* mice and further crossed with *CBF:H2B-NVenus* (Venus) mice for retinal harvest at P5. (D) High magnification image of retinal vasculature stained for αSMA (white) and Erg1 (blue). Expression of Venus is in green. Dashed lines outline vSMCs. Yellow arrows indicate vSMCs expressing Venus and white arrows highlight vSMCs that lack Venus expression. Scale bars, 35µm. (E) Quantification of Venus expression in vSMCs of control and Rbpj$^{i\Delta SMC}$ mice, validating efficient recombination of *Rbpj* in *Acta2-CreER^{T2}* mice and the loss of Notch signaling in vSMCs (n = 3-4). Data was analyzed using unpaired two-tailed test with Welch's correction.* $p < 0.05$.

SUPPLEMENTAL FIGURE 6

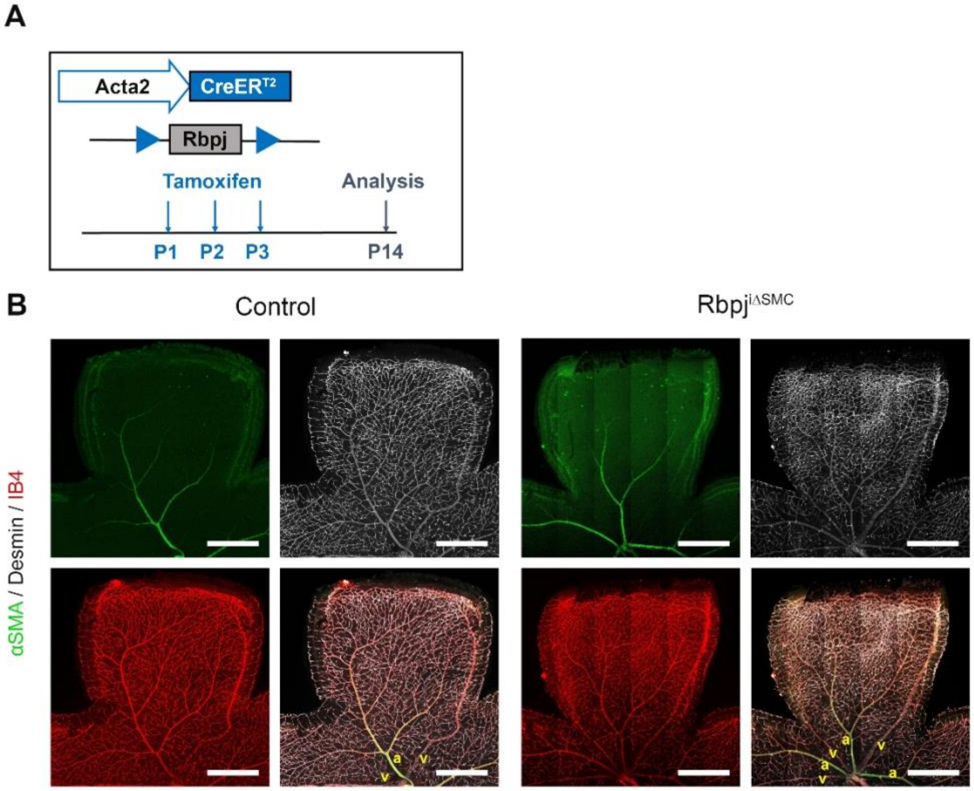

Supplemental Figure 6. Deletion of smooth muscle cell-specific Notch signaling does not result in AVMs. (A) Diagram of smooth muscle cell-specific inhibition of Notch signaling perinatally using *Acta2-CreRT^{T2}* mice and their analysis at P14. (B) Confocal images showing retinal vasculature stained with IB4 (white), NG2 (red) for pericytes and alpha-smooth muscle actin (αSMA, green) for vascular smooth muscle cells. a = artery and v = vein. n = 3 control and 8 Rbpj$^{i\Delta SMC}$ mice. Scale bars, 600 µm.

SUPPLEMENTAL FIGURE 7

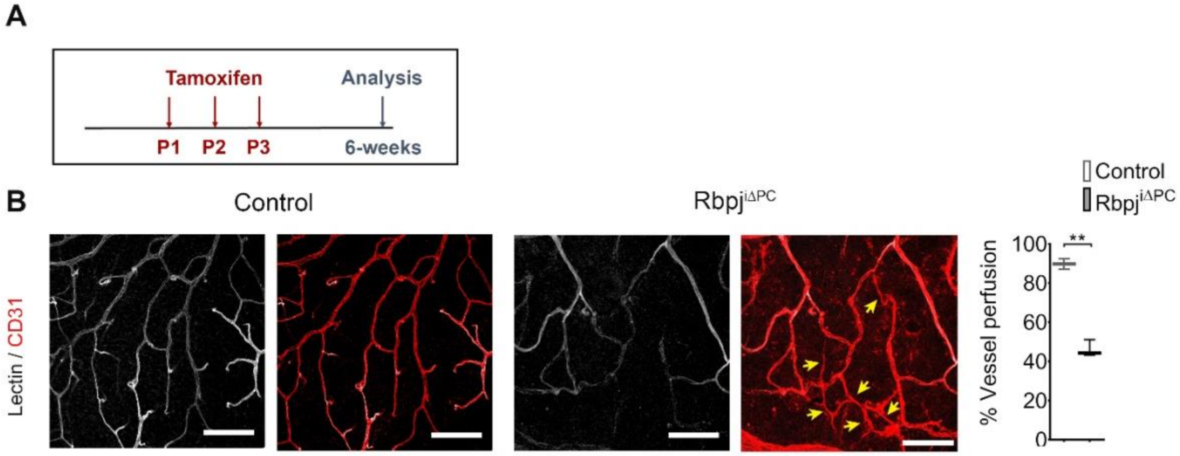

Supplemental Figure 7. Poor vessel perfusion observed in Rbpj$^{i\Delta PC}$ mice. (A) Diagram of tamoxifen administration to postnatal control and Rbpj$^{i\Delta PC}$ mice and analysis at 6-weeks. (B) Images and quantification of lectin perfused retinas, with Tomato Lectin (white) and CD31 (red) to label the endothelium. Arrows highlight non-perfused vessels (Lectin$^-$, CD31$^+$). Quantification of % vessel perfusion in control (n = 2) and Rbpj$^{i\Delta PC}$ mice (n = 3). Data was analyzed using unpaired two-tailed test with Welch's correction. ** $p < 0.01$.

SUPPLEMENTAL FIGURE 8

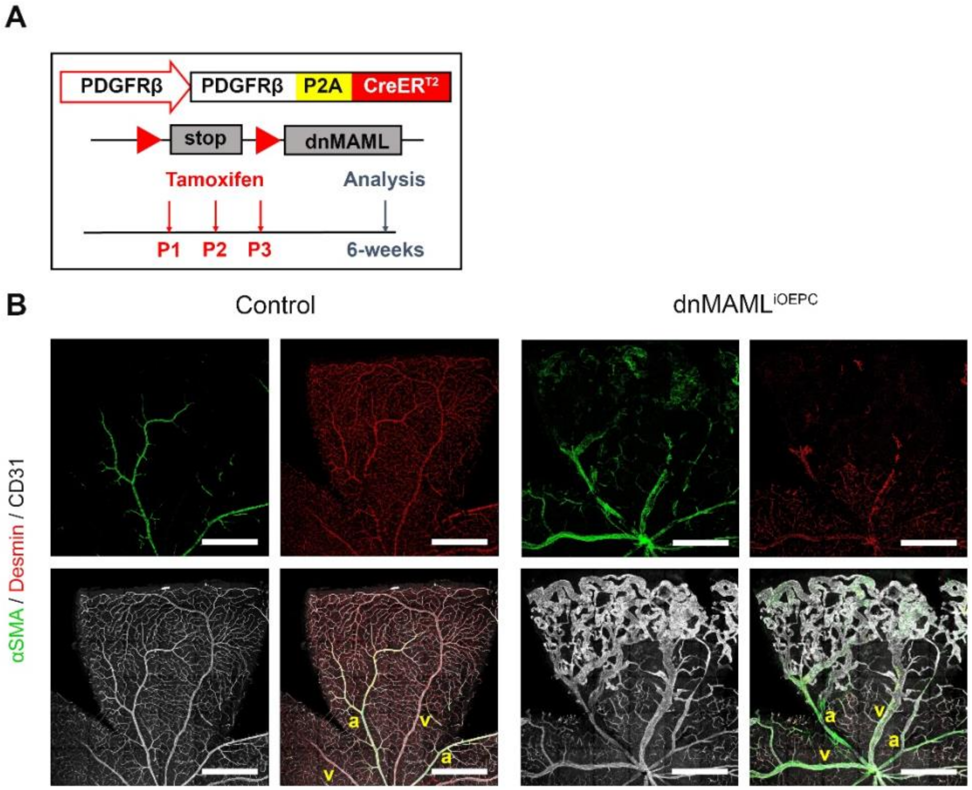

Supplemental Figure 8. Inhibition of Notch signaling in pericytes by overexpression of dnMAML recapitulates AVM phenotype observed in Rbpj$^{i\Delta PC}$ mice. (A) Diagram of perivascular inhibition of Notch signaling via dominant negative MAML (dnMAML) overexpression and their analysis at 6-weeks. (B) Confocal images showing 6-week retinal vasculature stained with anti-CD31 (white), anti-Desmin (red) for pericytes and anti-smooth muscle actin (αSMA, green) for vascular smooth muscle cells (vSMCs). a = artery and v = vein. n = 7 controls and n = 5 dnMAML^{iOEPC} mice. Scale bars, 600 μm.

SUPPLEMENTAL FIGURE 9

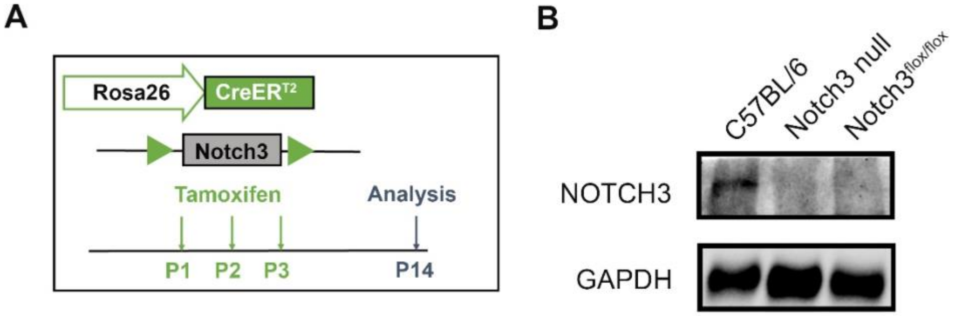

Supplemental Figure 9. Validation of tamoxifen-inducible deletion of NOTCH3. (A) Diagram of tamoxifen administration to postnatal *ROSA26-CreER*^{T2}*; N3*$^{flox/flox}$ mice and analysis at P14. (B) Western blot for Notch3 and Gapdh (loading control) on whole retinal lysates from C57BL/6 control mice, Notch3 null mice and *ROSA26-CreER*^{T2}*; Notch3*$^{flox/flox}$ tamoxifen-treated mice.

SUPPLEMENTAL FIGURE 10

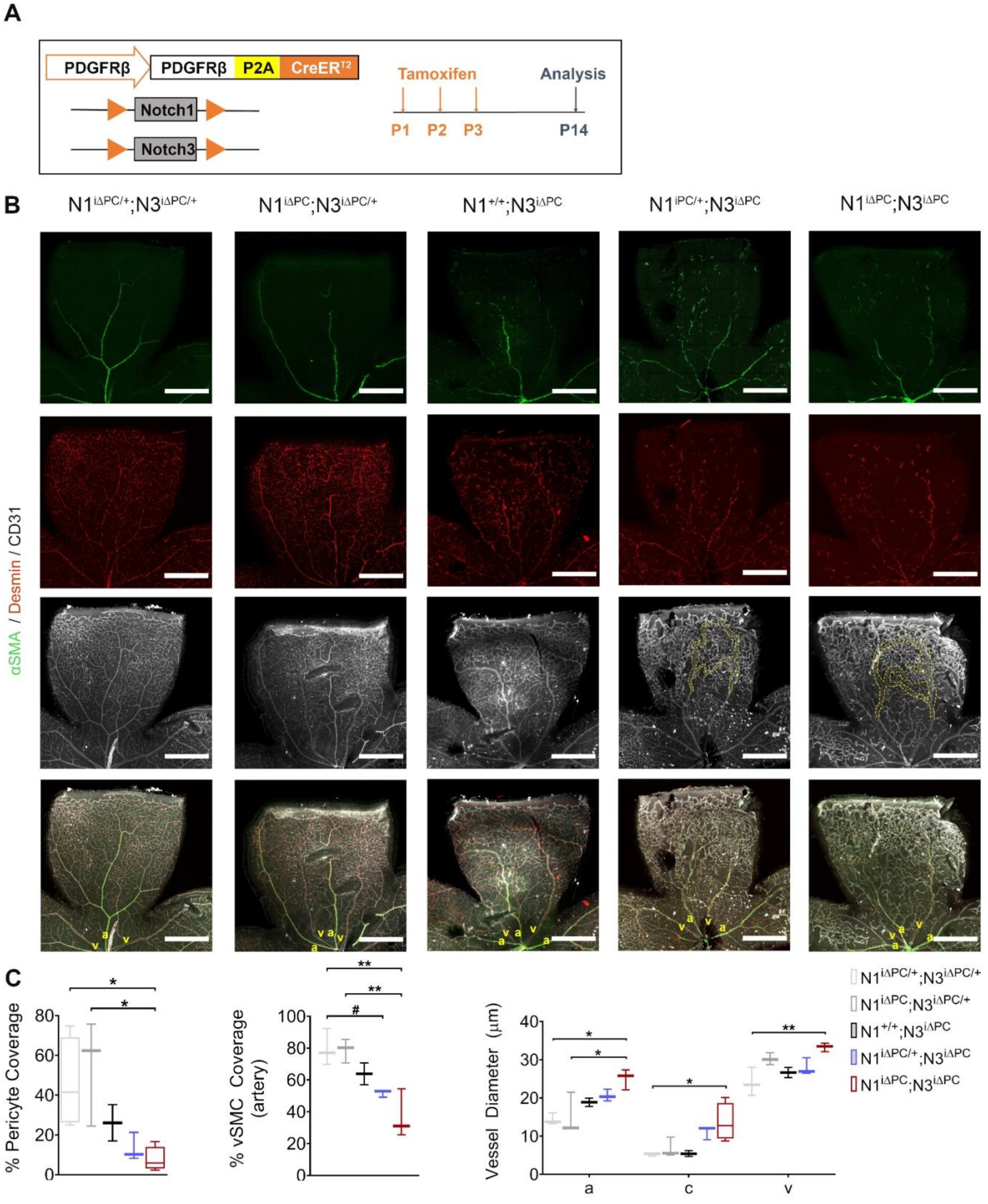

Supplemental Figure 10. Combined loss of Notch1 and Notch3 in pericytes results in AVM formation. (A) Diagram of tamoxifen administration to postnatal control and mice deficient for pericyte-Notch1 and Notch3 receptors and analysis at P14. (B) Confocal images showing the retinal vasculature stained with anti-CD31 (white), anti-Desmin (red) for pericytes and anti-smooth muscle actin (αSMA, green) for vascular smooth muscle cells (vSMCs). Note the abnormally enlarged AV connections and irregular distribution of perivascular cells in mutant mice with increasing deficiency of pericyte-Notch1 and Notch3 receptors. Yellow dashed lines highlight enlarged arteriovenous connections. Scale bars, 600 µm. (C) Quantification of % pericyte coverage, arterial vSMC coverage and vessel diameter (n = 2-5). Data was analyzed using one-way ANOVA with Tukey post-test. a = artery, c = capillary and v = vein. * $p < 0.05$, # $p = 0.052$.

SUPPLEMENTAL FIGURE 11

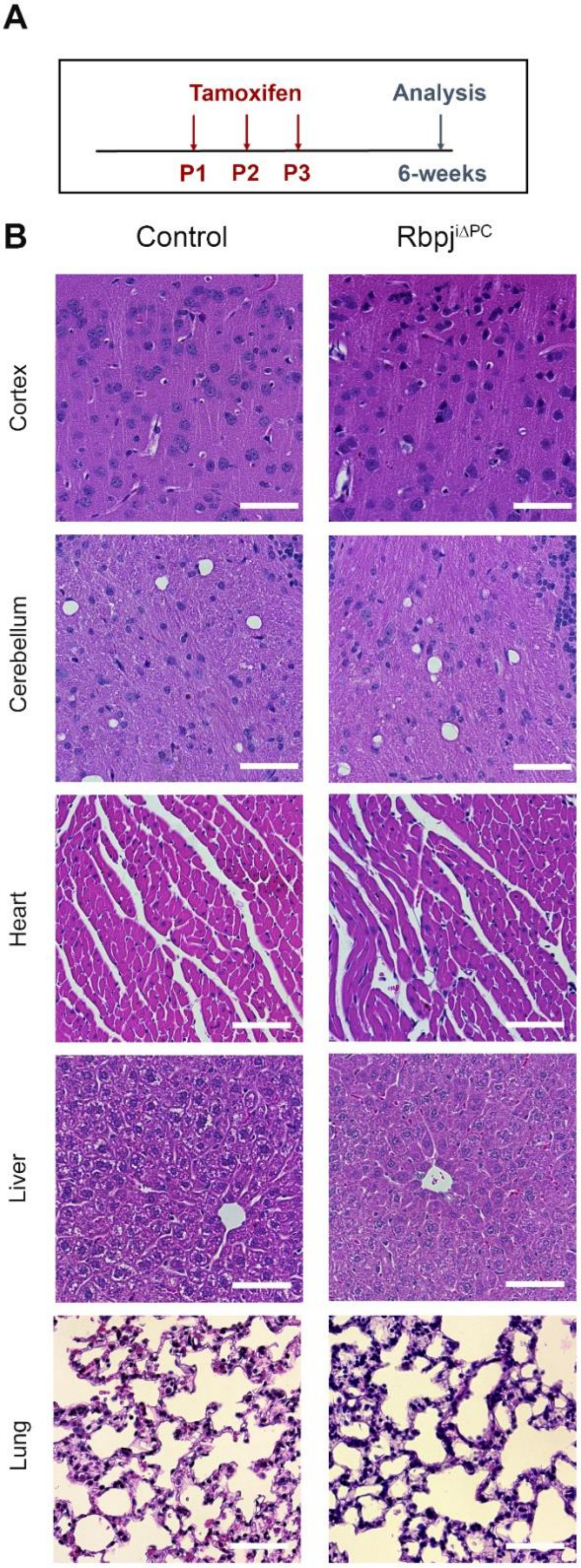

Supplemental Figure 11. Absence of vascular abnormalities in brain, heart, liver and lung 6 weeks after deletion of perivascular Notch signaling. (A) Diagram of tamoxifen administration to postnatal control and Rbpj$^{i\Delta PC}$ mice and analysis at 6-weeks. (B) Representative images of Hematoxylin and Eosin (H&E) stained sections from the brain (cortex and cerebellum), the heart, liver and lung of Rbpj$^{i\Delta PC}$ and control mice. n = 5. Scale bars, 65 µm.

SUPPLEMENTAL FIGURE 12

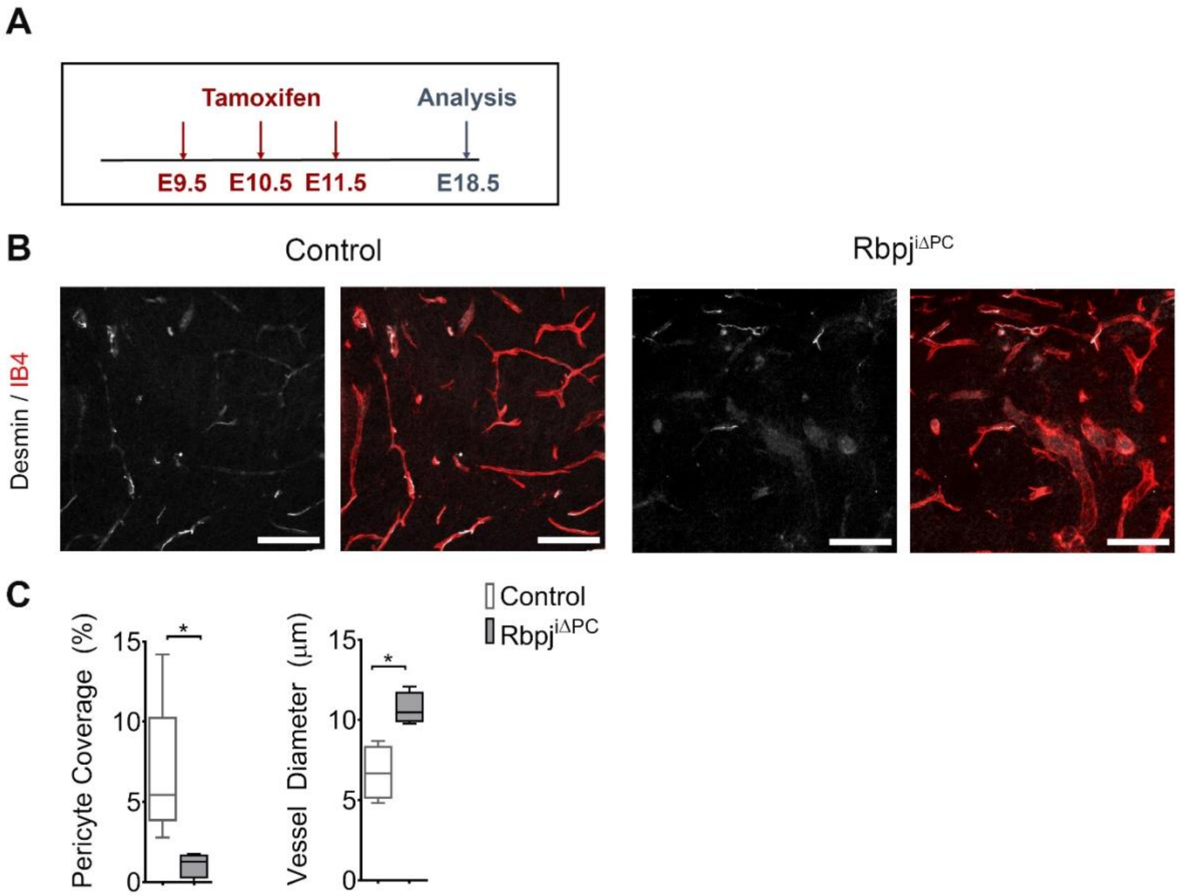

Supplemental Figure 12. Vascular malformations in the brain of Rbpj$^{i\Delta PC}$ embryos. (A) Diagram of tamoxifen administration to embryonic control and Rbpj$^{i\Delta PC}$ mice and analysis at E18.5. (B) High magnification images of the forebrain vasculature stained with anti-Desmin (white) for pericytes and Isolectin B4 (IB4, red) for the endothelium. Scale bars, 100 µm. Quantification of % pericyte coverage and vessel diameter in control and Rbpj$^{i\Delta PC}$ embryonic mice (n = 3-4). Data was analyzed using unpaired two-tailed test with Welch's correction. * $p < 0.05$.

SUPPLEMENTAL FIGURE 13

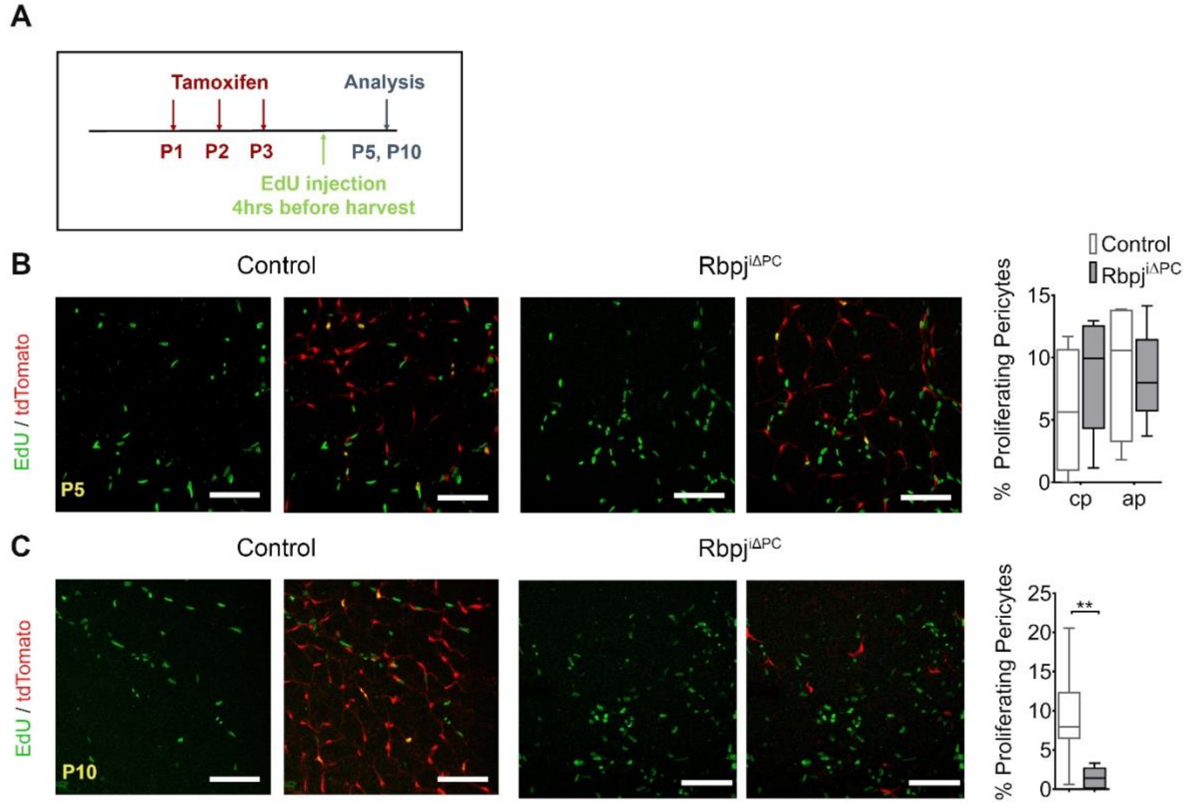

Supplemental Figure 13. Pericyte proliferation in mice with perivascular loss of Notch signaling. (A) Diagram of tamoxifen and EdU administration to postnatal control and $Rbpj^{i\Delta PC}$ mice and analysis at P5 and P10. (B) Representative images from the capillary plexus and quantification of P5 retinal vasculature of control and $Rbpj^{i\Delta PC}$ mice stained for EdU (green) with tdTomato reporter (red) utilized to highlight pericytes in control and $Rbpj^{i\Delta PC}$ vasculature. Scale bars, 100 µm. Quantification of % proliferating pericytes in the capillary plexus (cp) and angiogenic plexus (ap) of control and $Rbpj^{i\Delta PC}$ mice (n = 3-4). (C) Representative images and quantification of P10 retinal vasculature of control and $Rbpj^{i\Delta PC}$ mice stained for EdU (green) with tdTomato reporter (red) utilized to highlight pericytes in control and $Rbpj^{i\Delta PC}$ vasculature. Scale bars, 100 µm. Quantification of % proliferating pericytes in control and $Rbpj^{i\Delta PC}$ mice (n = 6-9). Data was analyzed using unpaired two-tailed test with Welch's correction. ** $p < 0.01$.
